# Transcontinental spread and evolution of *Mycobacterium tuberculosis* W148 European/Russian clade toward extensively drug resistant tuberculosis

**DOI:** 10.1038/s41467-022-32455-1

**Published:** 2022-08-30

**Authors:** Matthias Merker, Jean-Philippe Rasigade, Maxime Barbier, Helen Cox, Silke Feuerriegel, Thomas A. Kohl, Egor Shitikov, Kadri Klaos, Cyril Gaudin, Rudy Antoine, Roland Diel, Sonia Borrell, Sebastien Gagneux, Vladyslav Nikolayevskyy, Sönke Andres, Valeriu Crudu, Philip Supply, Stefan Niemann, Thierry Wirth

**Affiliations:** 1 Molecular and Experimental Mycobacteriology, Research Center Borstel, Borstel, Germany; 2 German Center for Infection Research, Partner site Hamburg-Lübeck-Borstel-Riems, Borstel, Germany; 3 Evolution of the Resistome, Research Center Borstel, Borstel, Germany; 4 EPHE, PSL University, Paris, France; 5 Institut de Systématique, Evolution, Biodiversité, ISYEB, Muséum national d’Histoire naturelle, CNRS, Sorbonne Université, EPHE, Université des Antilles, Paris, France; 6 Centre International de Recherche en Infectiologie, INSERM U1111, CNRS UMR5308, Université Lyon 1, ENS de Lyon, Lyon, France; 7 Division of Medical Microbiology and Institute of Infectious Disease and Molecular Medicine, University of Cape Town, Cape Town, South Africa; 8 Federal Research and Clinical Centre of Physical-Chemical Medicine, Moscow, Russian Federation; 9 SA TUH United Laboratories, Mycobacteriology, Tartu, Estonia; 10 Genoscreen, Lille, France; 11 Univ. Lille, CNRS, Inserm, CHU Lille, Institut Pasteur de Lille, U1019 - UMR 9017 - CIIL - Centre d’Infection et d’Immunité de Lille, F-59000 Lille, France; 12 Institute for Epidemiology, Schleswig-Holstein University Hospital, Kiel, Germany; 13 Lung Clinic Grosshansdorf, German Center for Lung Research (DZL), Airway Research Center North (ARCN), 22927 Großhansdorf, Germany; 14 Swiss Tropical and Public Health Institute, Allschwil, Switzerland; 15 University of Basel, Basel, Switzerland; 16 Imperial College London, London, UK; 17 National and WHO Supranational Reference Center for Mycobacteria, Research Center Borstel, Borstel, Germany; 18 National TB Reference Laboratory, Institute of Phthisiopneumology, Chisinau, Moldova

**Keywords:** Bacterial genetics, Tuberculosis, Phylogeny

## Abstract

Transmission-driven multi-/extensively drug resistant (M/XDR) tuberculosis (TB) is the largest single contributor to human mortality due to antimicrobial resistance. A few major clades of the *Mycobacterium tuberculosis* complex belonging to lineage 2, responsible for high prevalence of MDR-TB in Eurasia, show outstanding transnational distributions. Here, we determined factors underlying the emergence and epidemic spread of the W148 clade by genome sequencing and Bayesian demogenetic analyses of 720 isolates from 23 countries. We dated a common ancestor around 1963 and identified two successive epidemic expansions in the late 1980s and late 1990s, coinciding with major socio-economic changes in the post-Soviet Era. These population expansions favored accumulation of resistance mutations to up to 11 anti-TB drugs, with MDR evolving toward additional resistances to fluoroquinolones and second-line injectable drugs within 20 years on average. Timescaled haplotypic density analysis revealed that widespread acquisition of compensatory mutations was associated with transmission success of XDR strains. Virtually all W148 strains harbored a hypervirulence-associated *ppe38* gene locus, and incipient recurrent emergence of *prpR* mutation-mediated drug tolerance was detected. The outstanding genetic arsenal of this geographically widespread M/XDR strain clade represents a “perfect storm” that jeopardizes the successful introduction of new anti-M/XDR-TB antibiotic regimens.

## Abstract (web summary)

An outbreak of the multidrug-resistant Mycobacterium tuberculosis lineage W148 has spread widely across Russia, Central Asia and Europe. Here, the authors use whole genome sequences of ~700 isolates of this lineage collected over ~20 years to analyze its spread, evolution of drug resistance, and impact of compensatory mutations.

## Introduction

Bacteria of the *Mycobacterium tuberculosis* complex (Mtbc), the causative agent of tuberculosis (TB), caused 10 million new TB cases in 2019 and are the major cause of death worldwide due to antimicrobial resistance^1^. Infections with multidrug resistant (MDR) Mtbc strains, i.e., at least resistant to isoniazid [INH] and rifampicin [RIF], require treatment regimens based on combinations of at least four drugs for up to 2 years. This treatment often comes with severe side effects and cure rates hardly exceed 50% globally^2^. Close to half a million new RIF resistant/MDR-TB cases occur annually^1,3^. Overall, 10% of the MDR strains are estimated to be additionally resistant to at least one fluoroquinolone and one second-line injectable drug, classifying them as extremely drug resistant (XDR) or pre-XDR, according to previous or newly modified WHO definitions, respectively, and rendering treatment challenging or almost impossible in some cases.

While it was long assumed that MDR-TB was mostly arising from acquisition of drug resistance during ineffective TB treatment, modeling data suggest that incident MDR-TB is now globally driven by person-to-person transmission of MDR strains, i.e., by primary resistance^4^. Consistently, large MDR-TB outbreaks involving extensive transmission of MDR strains over several decades have been described in, e.g., KwaZulu Natal in South Africa^5,6^ and in Argentina^7^. However, despite this extended time scale and although the implicated strains are part of the otherwise globally successful Mtbc lineage (L4)^8^, these outbreaks have been essentially contained within the corresponding regions. A recent study covering 15 countries also showed minimal cross-border transmission of drug-resistant L4 strains, although this restriction was attributed to the relatively recent emergence of these strains^9^. These observations raise thus questions on the pace of drug resistance evolution and on the factors that may limit or drive (inter)national spread of highly drug resistant TB strains.

To address these questions, particular MDR-TB clones highly prevalent in the Russian Federation and several countries in Eastern Europe and Central Asia represent critical vehicles^10,11^. These world regions are particularly affected by the MDR-TB epidemic, with MDR-TB rates reaching levels of more than 20% among new patients^11^. This epidemic is largely linked to MDR strains of L2 (Beijing genotype), which have been proposed to acquire drug resistance more rapidly than L4 strains in vitro^12^. We recently showed that the success of L2 MDR outbreak strains in Uzbekistan was also related to the acquisition of compensatory mutations that mitigate the fitness cost of RIF resistance conferring mutations^13^. Sustained transmission of these outbreak strains was associated with additional accumulation of drug resistance, in a setting lacking comprehensive drug susceptibility testing at the time^14^.

Here, we investigate 720 isolates of a single MDR outbreak clade^11^ part of L2, referred to as W148 European/Russian, from 23 countries sampled between 1995 and 2013. W148 strains are the main contributors to the MDR epidemic in Russia and Eastern Europe, and since the USSR’s fall, have also propagated to Western Europe, likely driven by economic or medical migrations of TB patients. As such, this strain dataset represents a unique resource to study the longitudinal and geographical spread, transmission efficacy and drug resistance evolution of Mtbc strains under treatment pressure. Whole genome sequencing (WGS) and Bayesian demographic approaches were combined to infer the phylogenetic structure and origin, as well as bacterial demographic changes and evolutionary steps driving the rise and spread of M/(pre-)XDR-TB in Eurasia over the last decades.

## Results

### W148 phylogeography and spatio-temporal dispersion

**Fig. 1:**
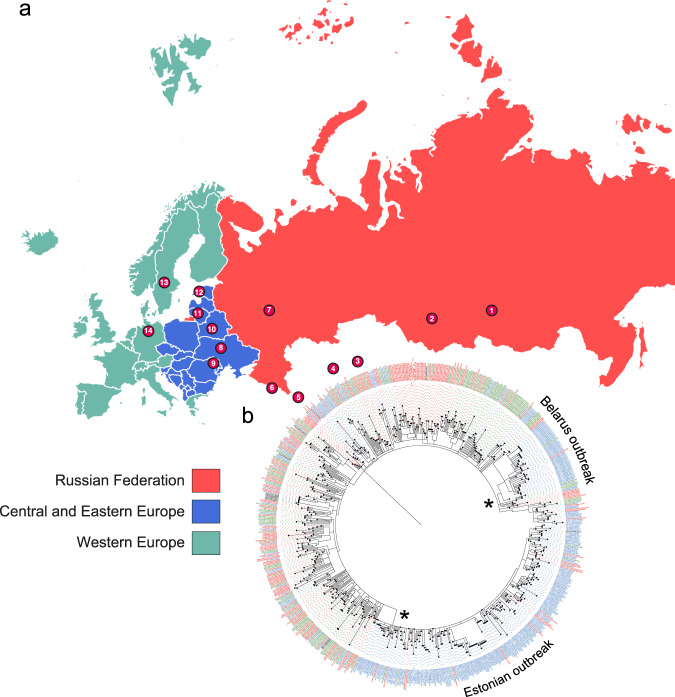
Sampling scheme of B0/W148 strains and phylogeographic expansion patterns in Eurasia. **a** Main locations (circled numbers and *N* ≥ 5) of sampled B0/W148 populations stratified to three principle geographic regions (1 Russian Federation Tuva, 2 Russian Federation central Asia, 3 Kazakhstan, 4 Uzbekistan, 5 Iran, 6 Abkhazia, 7 Russian Federation Samara, 8 Ukraine, 9 Republic of Moldova, 10 Belarus, 11 Lithuania, 12 Estonia, 13 Sweden, 14 Germany). **b** Maximum-likelihood phylogeny of 720 B0/W148 strains, with tips shaded by principle geographic origin. The stars are indicative of two major outbreaks. The exhaustive list of all samples can be found in Supplementary Data 1.

We included a total of 731 clinical isolates of L2 for WGS analysis. Of those, 720 belonged to the previously defined W148 European/Russian outbreak based on specific single nucleotide polymorphisms (SNPs)^11^. The majority of the collection was sampled during the 21st century and originated from 23 countries, which were later grouped to 14 geographical regions (Fig. 1a, Supplementary Fig. 1, Supplementary Data 1). Eleven non-W148 modern lineage 2 isolates were used as outgroup in subsequent phylogenetic analysis. To avoid the inclusion of homoplastic sites, mainly due to independent acquisition of identical resistance conferring mutations in our phylogenetic reconstructions, we removed 610 variants located in genes and intergenic regions associated with drug resistance and bacterial fitness (Supplementary Data 1). A concatenated sequence alignment of 3508 SNPs differentiating the isolates was used to build a maximum-likelihood tree in order to unravel the geographic pattern present in the dataset (Fig. 1). The tree revealed a distinction between two major W148 sublineages, bifurcating from the most basal node in the phylogeny, at the extremity of the branch derived from the root (Fig. 1b). Most Belarus and Estonian strains clustered in a single subclade, indicating local epidemic spread. This pattern of local epidemics was also apparent from a minimum spanning tree (Supplementary Fig. 2) where central nodes, containing several identical strain genomes from Estonia or Belarus, are surrounded by numerous variants differing only by 2–5 SNPs. In contrast, strains from Western Europe were randomly distributed in the network suggesting the importation of W148 strains from multiple different regions.

**Fig. 2:**
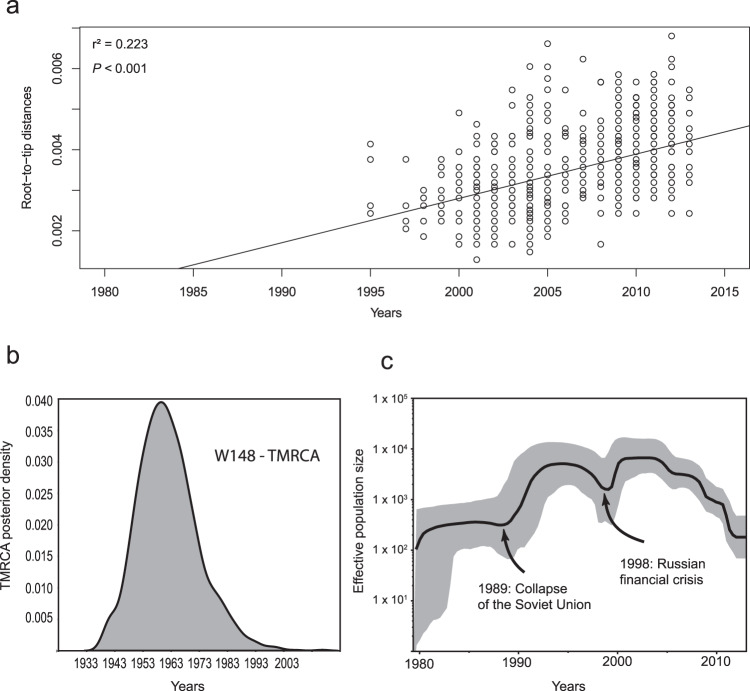
W148 is a measurably evolving population. **a** Linear regression analysis (least square approximation) showing correlation between root-to-tip distance and sampling years of the W148 strain collection (*n* = 720) covering the period 1995–2013. **b** Posterior density distribution of the W148 clade TMRCA. **c** Effective population size over time of the 720 W148 strains based on a Bayesian skyline approach using the HKY substitution model and a Log normal relaxed clock model estimated a mean mutation rate of 1.12 × 10^−7^ substitutions per nucleotide per year. Gray shaded area represents changes in the effective population size giving the 95% highest posterior density (HPD) interval with the black line representing the mean value.

In an initial step, we verified that the W148 clade is a measurably evolving population (Fig. 2a, Supplementary Fig. 3), as required for robust molecular clock calibration. The best fitting evolutionary model was obtained under a Bayesian skyline model (Table S1), resulting in a mutation rate of 1.1 × 10^−7^ mutation per site per year, with 57.61% of variation across the tree. The time tree (Supplementary Fig. 4) estimates the W148 MRCA around 1963 (95% HPD 1958–1968), and indicates a sharp geographic dispersion to all Eurasia from the early seventies onwards, followed by two main outbreaks in Belarus and Estonia in the late eighties. The coalescent-based demographic reconstructions indicated that the clonal population markedly expanded with a twentyfold increase in the late eighties-early nineties, and suggested a milder, fivefold increase (albeit with less certainty given large confidence intervals) in the late nineties-early 2000s (Fig. 2c).

### Genetic diversity among the W148 cluster

Pairwise SNP distances among W148 isolates were unimodal, with a median of 32 SNPs (Supplementary Fig. 5). This definitively translates into a recent and weakly structured sublineage, confirming its status of an outbreak clone. Two other low frequency distribution pairs can be seen, with median SNPs around 170 and 210. Those correspond to the mean pairwise distances observed between W148 strains and other L2 members, including basal strains from this lineage.

### W148 drug resistance evolution

**Fig. 3:**
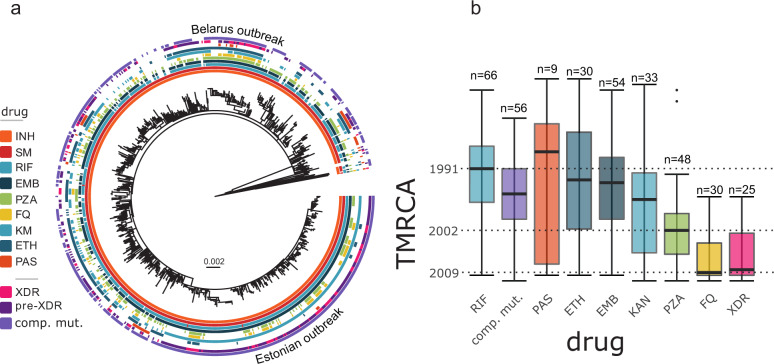
Antibiotic resistance profiles and emergence timing. **a** Bayesian time tree based on 5264 variable positions (SNPs) among the 720 W148 strains and 11 lineage 2 outgroup strains. The different crowns are showing the presence (filled box) or absence (empty box) of mutations conferring resistance to different drugs: INH Isoniazid, SM Streptomycin, RIF Rifampicin, EMB Ethambutol, PZA Pyrazinamide, FQ Fluoroquinolones, KM Kanamycin, ETH Ethionamide and PAS Para-aminoslicylic acid; strains drug resistance profiles: XDR, Pre-XDR and carrying putative *rpoB* compensation. **b** Box plot representing the emergence time of different antibiotic resistances. Highlighted dates indicate emergence of rifampicin resistance on top of pre-existing isoniazid resistance and thus MDR-TB (1991), pyrazinamide resistance (2002), fluoroquinolone resistance and XDR-TB (both 2009). Solid bars indicate median node ages, boxes represent the inter quartile range (IQR), whiskers extend to 1.5× IQR, outliers are shown as individual points.

All strains of the W148 clade were resistant against INH (*katG* S315T) and streptomycin (*rpsL* K43R), indicating that resistance to these drugs was already acquired by the W148 MRCA in 1963 (see above and Supplementary Data 1, Fig. 3a), which is consistent with the introduction of INH in TB treatment in the USSR in the 1960s. Further, rates of genotypic resistance were very high for other first-line drugs (Fig. 3a), i.e., RIF (93.2%), ethambutol (84.2%), pyrazinamide (49.6%), and overall high for second-line drugs, i.e., fluoroquinolones (22.4%), kanamycin (63.9%), amikacin (15.8%), capreomycin (16.0%), thioamide (44.4%), and PAS (8.9%) (Supplementary Data 1). Ten out of 720 W148 strains were identified with mutations mediating D-cycloserine resistance. We found no mutation previously described as associated with resistance against the newly introduced drugs delamanid and bedaquiline^15,16^. Alarmingly, among the 676 MDR W148 strains, 49.6% were classified as pre-XDR (either fluoroquinolone or second-line injectable drug resistance), and 20.9% were classified as XDR-TB, according to WHO definitions in place until early 2021 (Fig. S6 and Supplementary Data 1).

**Fig. 4:**
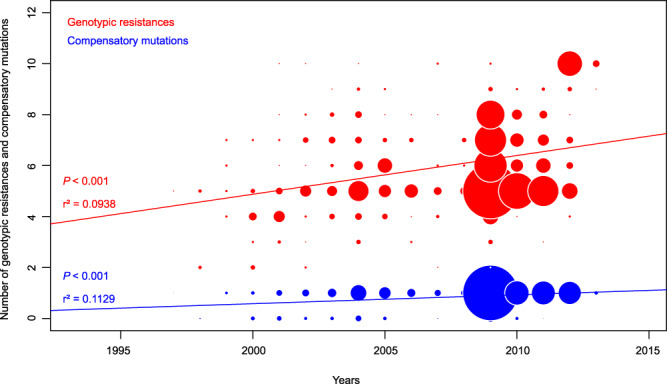
Bubble plot showing the number of genotypic resistances (in red) and compensatory mutations (in blue) as a function of strain’s years of isolation of all W148 strains (*n* = 720). Bubble sizes are proportionate to the number of strains. Regression analyses (least square approximation) were significant (*P* < 2.2 × 10^−16^) and regression curves are shown on the plot.

We then examined the dynamic and the chronological sequence of accumulation of resistance and compensatory mutations. By performing regression analyses, we detected a significant temporal signal in the accumulation of genotypic resistances (in red) and compensatory mutations (in blue) (Fig. 4), with respective *r²* = 0.09; *P* < 2.2 × 10^−16^ and 0.11; *P* < 2.2 × 10^−16^.

On average, RIF resistance mediating mutations in *rpoB* on top of pre-existing INH resistance and thus MDR, emerged around 1991 (inter quartile range (IQR) 1987–1997), in at least 66 independent events with one subsequent transmission event in the dated W148 phylogeny (Fig. 3b, Supplementary Fig. 6). This was followed by acquisition of an additional putative compensatory mutation in *rpoA*, *rpoB* or *rpoC* in 56 events around 1996 (IQR 1991–2000). Interestingly, acquisitions of resistance mutations to the second-line drug kanamycin (1997, IQR 1992–2006), but also PAS (1988, IQR 1983–2008) and ethionamide (1993, IQR 1985–2002) were also deeply rooted in the W148 phylogeny. Ethambutol and pyrazinamide resistance emerged around 1994 (IQR 1989–2000) and 2002 (1999–2006), respectively, rendering 331/720 (46%) W148 isolates fully first-line drug resistant (Supplementary Data 1). The last step toward (pre)-XDR genotypes was the acquisition of fluoroquinolone resistance in 2009 (IQR 2003–2010), in 25 independent events with subsequent transmission of the pre-XDR strain (Supplementary Fig. 6). Thus, on average W148 isolates evolved from MDR to (pre-)XDR within 20 years (Fig. 3b).

### Impact of compensatory mutations on transmission

We then focused on the impact of potential compensatory mutations on the transmission success of RIF resistant isolates (676/731 isolates), as judged by the genotype. Out of 676 RIF resistant isolates, 585 (86.5%) harbored a putative compensatory mutation in at least one of the three genes *rpoA, rpoB* or, mostly, *rpoC*. Only 9 RIF resistant isolates exhibited such mutations in two of these genes, and only one out of 55 (1.8%) RIF susceptible isolates harbored a mutation in either, *rpoA*, *rpoB* or *rpoC*, i.e., in this case *rpoC* N416N (Supplementary Data 1).

To disentangle the respective influences of drug resistance and compensatory mutations on the transmission success of W148 isolates, we used the timescaled haplotypic density (THD) method^17,18^.

**Fig. 5:**
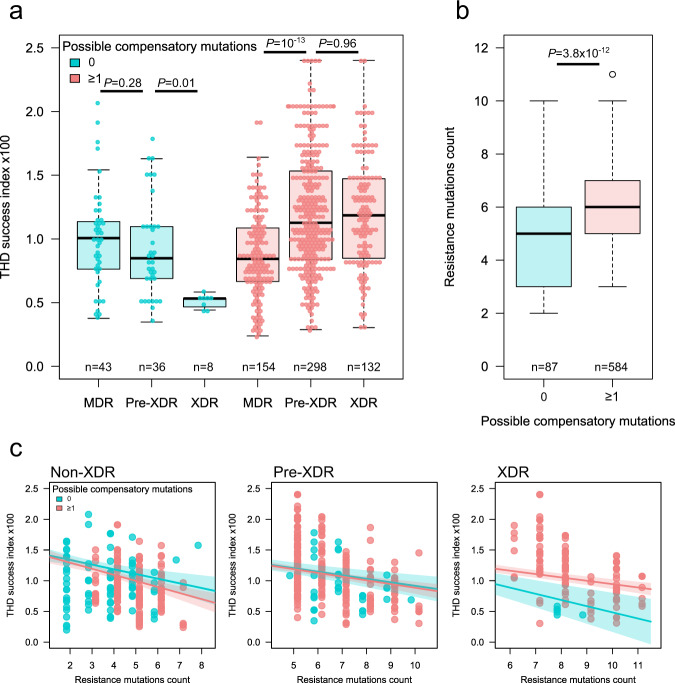
Compensatory mutations influence the epidemic success of drug-resistant variants of W148 *M. tuberculosis*. Whole genome sequences of 671 isolates were used to infer the presence of possible compensatory mutations and to compute indices of epidemic success using the THD method with a 10 y time scale. **a** Evolution of success indices with resistance status (MDR, and pre-XDR, XDR according to definitions in place in 2020) in isolates with and without possible compensatory mutations. The median success index decreased with resistance status in isolates lacking compensatory mutations and increased in isolates harboring these mutations. Solid bars indicate the median, boxes represent the inter quartile range (IQR), whiskers extend to 1.5× IQR. **b** The no. of resistance-conferring mutations per genome was higher in isolates harboring compensatory mutations. Solid bars indicate the median, boxes represent the inter quartile range (IQR), whiskers extend to 1.5× IQR, outliers are indicated by individual points. **c** The success indices decreased with the accumulation of resistance-conferring mutations within MDR, pre-XDR and XDR isolates. Colored lines and bands denote success indices (point estimate and 95% confidence interval, respectively) predicted by a linear model adjusted for population structure. In XDR, but not MDR and pre-XDR isolates, compensatory mutations were associated with a two-times slower decrease of success index per additional resistance mutation. All *P* values derived from two-sided Mann–Whitney U-tests.

For each isolate in a collection, the THD method considers the genetic distances of all other isolates to compute a measure of genotypic density that reflects the rate of divergence in the isolate’s ancestry, interpreted as a surrogate marker of epidemic success. The THD success indices are then used as response variables in regression models to enable correlating success with other strain characteristics^17,19,20^. Using linear mixed models (LMMs) adjusted for genetic population structure and country-level sampling variations (see Supplementary Note), we examined the associations of the success index with the resistance profile (MDR, and pre-XDR or XDR according to previous classification), the number of resistance-conferring mutations and the presence of compensatory mutations. When included independently in LMMs, neither the resistance profile nor the presence of compensatory mutations predicted success (*P* = 0.07 and 0.24, respectively, ANOVA *F-*tests). However, in a model including resistance status, compensatory mutations and their interactions, the addition of interaction terms improved model fit (*P* = 0.01, ANOVA *F*-test) and the presence of compensatory mutations was positively associated with THD in pre-XDR and XDR isolates (*P* = 0.04 and 0.006, respectively, coefficient *t*-tests). Hence, the relationship between resistance profile and epidemic success might be dependent on the presence of compensatory mutations. Indeed, while epidemic success decreased with increasing resistance profile (from MDR to pre-XDR and XDR) without compensatory mutations (Fig. 5a), the success increased from MDR to pre-XDR or XDR among isolates with compensatory mutations, showing that compensatory evolution, rather than increased resistance only, drives the success of highly resistant strains.

We then examined the impact of compensatory mutations on the incremental accumulation of resistance mutations, rather than crudely stratifying by MDR, pre-XDR or XDR profile. Resistance associated mutations were slightly but significantly more numerous in isolates with compensatory mutations compared to those without (median 6 vs 5 mutations, *P* = 8.1 × 10^−12^, Mann–Whitney U-test; Fig. 5b). The number of resistance mutations, when included in an LMM, was negatively associated with transmission success (*P* = 7.7 × 10^−5^), suggesting a global fitness cost upon gradual accumulation of resistance conferring mutations. To determine whether this fitness cost of gradual resistance accumulation was measurable independently of the resistance profile and/or compensatory mutations, we built a LMM comprising three-way interactions between the number of resistance mutations, resistance profile and compensatory mutations (Fig. 5c). The coefficients of these interactions were all negative, with individual *P* values ranging from 1.5 × 10^−12^ to 1.7 × 10^−7^. In MDR and pre-XDR isolates, the decrease of the success index associated with each additional resistance mutation was comparable in isolates with and without compensatory mutations (−1.4 × 10^−3^ vs −1.2 × 10^−3^ and −8.0 × 10^−4^ vs −8.6 × 10^−4^, respectively). In XDR strains, however, the decrease of success was 1.8-fold smaller in the presence of compensatory mutations (−6.5 × 10^−4^ vs −1.2 × 10^−3^). Within the MDR, pre-XDR and XDR groups, thus, the gradual accumulation of resistance mutations is consistently negatively associated with epidemiological success, but this association is attenuated by compensatory mutations in XDR isolates. Additional details of the models, along with complementary analyses using terminal branch lengths in the phylogeny as the model response variable in place of THD, can be found in the Supplementary Note file, Supplementary Fig. 7, Supplementary Fig. 8, and Supplementary Fig. 9.

### Targets of positive selection and hypervirulence-associated mutation

Lastly, we screened the W148 phylogeny for homoplasy, i.e., identical mutations occurring independently on different branches of the phylogeny and which cannot be explained by transmission events. Their independent evolution in the otherwise strongly clonal and genetically homogenous W148 outbreak would be a strong indication for positive selection. As a proof of principle, we re-introduced mutations in resistance mediating genes in the sequence alignment, which were previously excluded to calculate the ML tree. With the homoplasy analysis, we retrieved 77 such mutations in genes canonically implicated in drug resistance and compensatory effects (Supplementary Data 2). The remaining 82 mutations occurred in 47 genes with non-canonical or (as yet) unknown link with drug resistance or fitness compensation (Supplementary Data 2). Among the latter, 9 mutations were identified as associated with (multi-)drug resistance in previous genome-wide association studies^21–23^, comprising a *dnaA* chromosomal site recently shown to be associated with INH resistance^24^. Strikingly as well, 4 non-synonymous SNPs recurrently occurred in a total of 10 strains in the *prpR* (Rv1129c) gene. Mutations in these genes, including T131P and D160A, have recently been demonstrated to induce propionate metabolism-dependent tolerance to different drug classes in *M. tuberculosis*^25^. All but one of the concerned strains were (pre-)XDR, showing genotypic resistance to up to 10 anti-TB drugs, and harbored at least one compensatory mutation (Supplementary Data 1). Except for two Estonian strains sharing a same resistance profile, these *prpR* SNPs occurred in single, terminal tips of the phylogenetic tree, indicating that they were acquired concomitantly or after the last acquisition of resistance mutation.

In addition, we screened for the presence of an IS*6110* insertion-linked mutation in the *ppe38* gene, recently shown to be involved in hypervirulence of “modern” Beijing strains^26^. Consistent with previous findings^27,28^, we found this mutated configuration in all 731 genomes investigated, including both the W148 and the Beijing outgroup strains.

## Discussion

Using the largest genome set from a single outbreak of drug resistant *M. tuberculosis* ever investigated, we were able to report the transcontinental spread and evolution of an initial INH and streptomycin resistant clone toward extensive TB drug resistance over five decades. According to our dated phylogeny, this clone likely emerged in the early sixties. The geographical source was difficult to assess due to multiple population movements and a lack of sharp geographical structure in the ML tree, but Central Asia remains the most likely candidate region according to previous publications^11,28^. Two epidemic waves in the late 1980s and late 1990s coincided with social economic changes in the post-Soviet Era (respectively, the dissolution of Soviet Union and the 1998 Russian financial crisis) which likely also initiated subsequent westward expansions^29^. This continent-wide spread of M/XDR W148 strains as part of the MTBC lineage 2 is remarkable, as it contrasts with M/XDR *M. tuberculosis* clones part of other MTBC lineages, for instance L4.3.3 and L4.1.2.1, which were extensively transmitted over similar time scales but remained essentially contained in KwaZulu Natal in South Africa^5,6^ or in Argentina^7^. Likewise, global analysis of a large genome set of L4 strains showed that multiple independent drug resistant clones emerged at nation levels across 5 continents since the antibiotic era, but remained geographically restricted, with almost no cross-border transmission^9^.

Our data suggest a combination of bacterial genetic and human history factors as driving factor of this distinctive epidemiological success. While the spatial propagation of the parental clone began during the late-seventies, the apparent 20-fold increase in the W148 effective population size and the start of the epidemic spread, especially prominent in Estonia and Belarus, in the late 80 s temporally correlates with the fall of the Soviet Union and the collapse of its public health system. This historical episode is known to coincide with a 50% increase in TB mortality rate^29^, as well as epidemic peaks of other diseases such as diphtheria^31^ and syphilis^32^ in Eastern Europe and Russia at the same period. As a likely consequence of the poor TB management at the time^33^, and consistently with the predictable increase in mutation frequency upon increasing population size, we found that most drug resistances and putative compensatory mutations became fixed in the bulk of W148 strains around or following the expansion period in the 1990s.

Our results also point to the crucial role of compensatory evolution in determining the epidemic success of the W148 clade. In the evolutionary history of W148 strains, known compensatory mutations in the RNA-polymerase genes *rpoA*, *rpoB* and *rpoC*^34,35^ consistently appeared after the fixation of canonical RIF resistance conferring mutations in *rpoB*. Moreover, we found that W148 strains harboring at least one putative compensatory mutation were resistant to more drugs and exhibit higher transmissibility indices relatively to their counterparts devoid of such mutations, thus further coherent with effective mitigation of resistance related fitness cost and determinant of transmissibility. Our findings thus extend at a global scale recent conclusions on the role of compensatory mutations as drivers of transmission of MDR-TB strains in carceral and general populations in Georgia^36^. Interestingly, the expansion of the so-called LAM4/KZN XDR clone (part of L4) in KwaZulu-Natal likewise started in the early 90 s after the acquisition of presumed compensatory mutations in *rpoB*, but essentially remained limited to southern Africa since then, with only exceptional documented spill-over^37^, suggesting that other factors must have also contributed to the outstanding international spread of W148 strains.

The acquisition of extensive drug resistance and compensatory mutations specifically on top of an already underlying hypervirulent genetic background is likely part of the explanation, as it was already observed for other major pathogens^30,38,39^. Indeed, the W148 clade is a branch of the so-called “modern” Beijing sublineage L2, which has recently been shown to be characterized by an IS*6110*-linked deletion of the *ppe38* gene locus, leading to loss of secretion of substrates of the ESX-5 type VII secretion system and involved in a hypervirulent phenotype^26^. Our analysis confirmed the presence of such *ppe38* configuration in all 731 dataset strains. Moreover, the W148 clade additionally underwent two large scale genomic re-arrangements^40^, and has a characteristic frameshift mutation in the two component regulatory system KdpD and KdpE^11^, implicated in the response to oxidative stress^41^. A KdpDE deletion mutant of *M. tuberculosis* was shown to cause higher virulence in a mouse model^42^. Furthermore, we detected recurrent instances of emergence of *prpR* mutations mediating multidrug tolerance^25^ in terminal branches of our W148 phylogeny, corresponding in most cases to (pre-)XDR strains carrying both compensatory mutations and resistance mutations to up to 10 anti-TB drugs. Thus, the genetic make-up of the W148 clade may represent a unique “perfect storm” providing extended resistance with little to no fitness cost and incipient additional multidrug tolerance, on top of hypervirulence likely favoring rapid disease progression and enhanced infectiousness. Drug tolerance undermines overall antibiotic efficacy and favors additional drug resistance emergence^25^, and may further imperil sustained effectiveness of newly introduced anti-TB drugs, especially bedaquiline and linezolid, as resistance to either of these drugs and to a fluoroquinolone is now defining XDR. Moreover, the observed ongoing gain of drug tolerance associated mutations in strains that are already highly resistant and highly transmissible—up to transcontinental level—is of particularly serious concern.

Finally, host population movements over the time period have most probably further potentialized these exceptional bacterial features and contributed to the geographic expansion of the W148 clade. Our TMRCA estimate of the W148 origin was determined to the early nineteen sixties, followed by a first expansion to European Russia, and consistent with important population movements from West Siberia starting in the 1960s till the 1980s^28^. Subsequent expansion was most likely fueled by the known important migration further westward^43^, as well as by many patients suffering from W148 strain-caused M/XDR-TB and seeking medical care in Western Europe, which occurred and still occurs since the fall of the Berlin Wall. Nevertheless, and importantly, for the strain set isolated in Western European countries, one-third of the patients whose origin was documented were Western European-born, indicating thus effective allopatric transmission beyond populations of origin and further supporting the contribution of intrinsic bacterial features as well.

In conclusion, our results provide new insights into bacterial and host-related factors involved in the propagation at continental scale of an extensively drug resistant strain of *M. tuberculosis* over the last five decades. From a clinical and public health point of view, our population genomic approach reveals that most W148 strains have become resistant to multiple first- and second-line anti-TB drugs, including some that are potentially gaining additional multidrug tolerance. Extensive drug susceptibility testing and a tightly controlled programmatic introduction of newer anti-TB drugs and newly composed regimens will be critical to contain and prevent further emergence, spread and additional resistance amplification of XDR strains. Furthermore, we show how Bayesian approaches can detect fine scale TB expansions linked to past deficiencies in Public Health systems or economic crises. Recently, numerous disruptions in medical services caused by COVID-19 translated into a worrying reduction in TB case notifications (WHO 2019). Applying a similar strategy with TB strains collected from 2019 onward will likely allow unraveling and measuring the impact of the COVID-19 epidemics on TB, facilitating therefore local decision-making and prioritization of aid.

## Methods

### Data collection and whole-genome sequencing

The study comprises Mtbc strains obtained for routine diagnostic procedures. No additional patient material was obtained. Ethical approval was granted by the ethic commission of the University of Lübeck, Germany. Overall, 731 MTBC isolates were identified based on characteristic MTBC genotyping patterns classifying them as W148 Beijing family. This definition includes isolates with mycobacterial interspersed repetitive units (MIRU) genotype “100-32”, as well as a characteristic IS*6110* restriction fragment length polymorphism (RFLP) banding pattern described previously by Bifani et al.^44^. Both MIRU genotype 100-32 and the characteristic RFLP banding pattern have been linked with strains previously designated as “the successful Russian clone”^28^. Following WGS analysis (see below), 720/731 isolates had specific genetic polymorphisms classifying them unambiguously as W148 Europe/Russian outbreak clade^11^. This clade has been recently also termed L2.2 M4.5^45^ and clade B in a study in Samara, Russia^13^.

Isolates were sampled in 23 different countries between 1995 and 2013 and later grouped in 14 geographical regions. For the geographic assignment of the strains, the following rule was applied: prioritize the “patient origin”, if not available implement “the country of isolation”. All samples belonging to Eurasia with more than five strains are represented on the geographical map (Fig. 1a). Other, marginal samples, from the Middle East and Africa, with <5 strains were not included in statistical analyses due to small samples sizes, but can be found in Supplementary Data 1. A detailed overview of the sampling per country and estimates for the regional prevalence of W148 strains are given in Supplementary Data 3, and numbers of MDR/XDR isolates per country are displayed in Supplementary Fig. 10. The global dataset entails 537 newly sequenced genomes, plus another 194 publicly available W148 genomes (see Supplementary Data 1). WGS was performed on all isolates using Illumina Technology (MiSeq and HiSeq 2500) with Nextera XT library preparation kits as instructed by the manufacturer (Illumina, San Diego, CA, USA). The raw data (fastq files) were submitted to the European nucleotide archive (accession numbers are given in Supplementary Data 1).

### Mapping of reads, SNP filtering and genotypic drug resistance prediction

Fastq files/reads were mapped to the *M. tuberculosis* H37Rv genome (GenBank ID: NC_000962.3) with the MTBseq pipeline as described earlier^46^. Briefly, variants were called with a minimum coverage of 10 reads and at least 75% allele frequency. After exclusion of drug resistance associated genes, repetitive regions and non-informative/non discriminating SNPs, the remaining positions that matched the above-mentioned threshold levels in at least 95% of all isolates were considered as valid and used for all isolates in a concatenated sequence alignment.

To determine strain genotypic drug resistances, we first considered all mutations in 28 genes implicated in resistance against first- and second-line drugs as well as *rpoA* and *rpoC* associated with compensatory effects (Supplementary Data 4). In the context of this study, we only considered a mutation in either *rpoA*, *rpoB*, or *rpoC*, as putative compensatory mutation when it co-occurred with a RIF resistance conferring mutation. We excluded known phylogenetic variants for the resistance prediction according to ref. 47. Rationales and references for mutations considered as resistance conferring are given in Supplementary Data 4. Homoplasy was analyzed with HomoplasyFinder (accessed at 26.10.2020, https://github.com/JosephCrispell/homoplasyFinder) as described earlier^48^. As input, we used the sequence alignment described above and re-introduced SNPs in genes implicated in drug resistance and compensatory effects, and a maximum-likelihood tree based on a sequence alignment excluding genes associated with drug resistance and compensatory evolution (see below).

### Time-dependent phylogenetic and phylogeographic reconstruction

Phylogenetic relationships were reconstructed using the maximum-likelihood approach implemented in Phyml 3.412^49^. The robustness of the maximum-likelihood tree topology was assessed with bootstrapping analyses of 1000 pseudoreplicated datasets. Phylogenies were rooted with the midpoint rooting option using FigTree software v1.4 and with the reference *M. tuberculosis* strain H37Rv, both resulting in the same topology. The profile of drug resistances for each strain and information of compensatory mutations were plotted on the maximum-likelihood tree using Itol^50^. Linear regression analysis of the root-to-tip distances against sampling time was performed using TempEst1.5^51^. To assess the robustness of our root-to-tip regression, we performed a permutation test of 5000 replicates using the lmPerm Package 2.1.0^52^ in R. As a complementary assessment of the temporal signal in the data, date randomization was performed on our datasets using the TipDatingBeast 1.1.0 R package^53^. Sampling dates of the genomes were randomly shuffled five times, and date-randomized datasets were analyzed with Beast V2.3.2 using the same parameters as the original ones. For all simulations, there was no overlap between the substitution rates 95% HPD intervals between the real data and the randomized data, suggesting that the data contain sufficient temporal structure and spread.

For the coalescent-based analyses, evolutionary rates and tree topologies were analyzed using the general time-reversible (GTR) and Hasegawa–Kishino–Yano (HKY) substitution models with gamma distributed among-site rate variation with four rate categories (Γ4). The substitution rate was estimated under different demographic and clock models using Beast v2.3.2^54^ taking advantage of a sampling timeframe from 1995 to 2013. We tested both a strict molecular clock (which assumes the same evolutionary rates for all branches in the tree) and a relaxed clock that allows different rates among branches. Constant-sized and Bayesian skyline plot models, based on a general, non-parametric prior that enforces no particular demographic history were used. For each model, two independent chains were conducted for 100 million generations and convergence was assessed by checking ESS values for key parameters using Tracer v1.6. We used Tracer v1.6 to calculate the log_10_ Bayes factors in order to compare the models after a burnin of 10% of the chain. Bayes factors represent the ratio of the marginal likelihood of the models being compared. Approximate marginal likelihoods for each coalescent model were calculated via importance sampling (1000 bootstraps) using the harmonic mean of the sampled likelihoods. A ratio between 3 and 10 indicates moderate support that one model better fits the data than another, whereas values >10 indicate strong support.

In addition, to compare regions of different sample sizes we calculated the nucleotide diversity *pi* per regions and performed a rarefaction procedure to correct for sample size differences, using bootstrapping and subsampling of 19 individuals (corresponding to the smallest population size considered in the analysis, i.e., from Lithuania) with 10,000 repetitions, in R, using the package Ape^55^ and Pegas^56^. The minimum spanning tree was produced using BioNumerics version 7.6.

### Statistical analysis, THD success index computation and modeling

The THD success index was computed as described elsewhere^17^ based on the matrix of genetic distances between isolates (SNPs counts). User-defined parameters were a mutation rate of 1.1 × 10^−7^ mutation per site per year, an effective genome size (number of positions retained for SNP calling) of 3.97 × 10^6^ and a time scale of 10 y, consistent with the relatively fast pace of expansion of W148 suggested by demographic analyses (Fig. 2c).

To account for variations in the sampling effort between countries in our dataset, the THD computations were corrected for sampling bias. The THD reflects both the abundance and genetic proximity of isolates in a group of interest, so that an unbalanced sampling coverage between groups can falsely increase THD in groups with higher coverage. Consider the following example, where two genetically distinct groups A and B have similar demographic history such that their true average THD values are equal, and their THD estimates depend on their respective sample sizes. If, due to unbalanced sampling effort, individuals in A and B were sampled with a probability (sampling coverage) of 1.0 and 0.5, respectively, we expect THD estimates in A to be higher than in B due only to sampling bias. Because each THD value is an average of densities, this sampling bias can be corrected for by using a weighted average, with weights inversely proportional to sampling coverage. In our example, assigning weights of 1 to individuals in A and weights of 2 to individuals in B would restore the balance between A and B. Based on this rationale, we estimated the sampling coverage for each country in our dataset (Supplementary Data 3) to assign weights to isolates during THD computations. The THD success index was then compared between groups using Mann–Whitney U-test or used as the response variable in LMMs. All constructed LMMs were controlled for population structure and country-specific variations (see details below). The significance of LMM coefficients was assessed using package lmerTest v3.1-3. All THD computations used R software version 4.0.2. Detailed model outputs can be found in the Supplementary Note. R scripts and data for all THD models and figure generation can be found at https://github.com/rasigadelab/xdrtb_thd and at the Zenodo repository at https://zenodo.org/record/6826135 under DOI 10.5281/zenodo.6826135.

Correction for population structure in THD models is used to detect potential predictors independent of vertical inheritance^18^. Correction for population structure was conducted using principal component analysis^57^. Genetic principal components are distance-preserving coordinates in the space of genetic distances (SNP differences) between isolates, the same space over which THDs are computed. To correct for population structure in THD regression models, we introduced genetic principal components as continuous, fixed-effect control variables. Doing so introduces the location of each isolate in the population structure as a model predictor. If THD indices only depend on population structure but not on other predictors such as drug resistance, the addition of these other predictors will not improve model fit and these predictors will not be considered independent. Conversely, if other predictors contribute to THD prediction independent of population structure (typically if their effect is seen among strains with independent arisal in the phylogeny), their addition along with genetic principal components will improve model fit and these predictors will be considered independent of the population structure.

The genetic principal components were computed as follows. First, a reasonably large set (*n* = 20) of genetic principal components were computed from the matrix of genetic distances using classical metric multidimensional scaling (standard R command cmdscale). We did not use the full set of genetic principal components as controls in the THD mixed-effect models, as this would lead to increased noise and diminished statistical power. To reduce noise, we selected a subset of the genetic principal components most strongly associated with THD, to be used as controls in THD mixed-effects models. To select the subset of relevant genetic components, a linear regression of THD was constructed, of the form [Eq. 1]

1 $$y={Gg}+\epsilon$$

where $y$ is the known vector of THD indices; $G$ is the known matrix of genetic principal components; $g$ is the unknown vector of coefficients associated with the genetic principal components; and $\epsilon$ is the error term, assumed to follow a centered normal distribution. This full model was used as input to a stepwise variable selection procedure in which genetic principal components are added or removed at each step while monitoring model fit. The procedure stops when complexity-penalized model fit (as estimated using Akaike Information Criterion) is not strengthened anymore by adding or removing genetic principal components (standard R command step). The subset of genetic principal components (*n* = 4, namely, components 1, 2, 3 and 7) in the final, best-fitting model (adjusted *R²* = 0.29) were retained and used as fixed-effect control variables in THD mixed-effect models. The same approach was used in alternative models using terminal branch lengths in place of THD indices as the response variable (see Supplementary Methods).

Linear mixed models (LMMs) using THD indices as the response variable had the following structure [Eq. 2],

2 $$y=X\beta+C\gamma+{Zu}+\epsilon$$

where $y$ is the known vector of THD indices; $X$ is the known matrix of predictors of interest as indicated in text, such as the resistance group; $\beta$ is the unknown vector of fixed-effect coefficients associated with the fixed-effect predictors; $C$ is the known matrix of fixed-effect controls, typically the set of genetic principal components used to control for population structure (see above); $\gamma$ is the unknown vector of coefficients associated with the fixed-effect controls; $Z$ is the known matrix of random-effect controls, namely the geographic region coded as a categorical variable; $u$ is the unknown vector of coefficients associated with the random-effect controls; and $\epsilon$ is the error term, assumed to follow a centered normal distribution.

### Impact of resistance-conferring and compensatory mutations on transmission success

We used multiple linear regressions to examine the respective contributions of antimicrobial resistance and putative fitness cost-compensating mutations to the transmission success of TB. To take transmission duration into account, we computed, for each isolate and each period length T in years (from 1 to 40 y before sampling), a transmission success score defined as the number of isolates distant of less than *T* SNPs, divided by *T*. This approach relied on the following rationale: based on MTBC evolution rate of 0.5 mutation per genome per year, the relation between evolution time and SNP divergence is such that a cluster with at most *N* SNPs of difference is expected to have evolved for approximately *N* years. Thus, transmission success score over T years could be interpreted as the size of the transmission network divided by its evolution time, hence as the average yearly increase of the network size. For each period *T*, the transmission success score was regressed on the number of resistance mutations and on the presence of putative compensatory mutations. The regression coefficients with 95% confidence intervals were computed and plotted against *T* to identify maxima, that is, time periods when the transmission success was maximally influenced by either resistance-conferring or - compensating mutations.

### Detection of genes under positive selection and IS6110 insertion-linked *ppe38* mutation

Homoplasies, likely signatures of positive selection, were identified using HomoplasyFinder^48^. IS*6110* insertions in the *ppe38* gene locus were screened in the 731 genomes by a specific pipeline, first including collection of IS*6110* sequence parts-containing reads identified by bowtie 2^58^ -based mapping on a IS*6110* reference sequence. Using the same mapping tool or the CLC Genomic Workbench software (Qiagen), collected reads were mapped on a *M. tuberculosis* H37Rv genome reference, from which all IS*6110* copies were excised in silico, to identify orthologous genome sequences flanking IS*6110* insertions in each isolate, as described in Antoine et al.^59^.

### Reporting summary

Further information on research design is available in the Nature Research Reporting Summary linked to this article.

## Supplementary information

Supplementary Information

Description of Additional Supplementary Files

Supplementary Data 1

Supplementary Data 2

Supplementary Data 3

Supplementary Data 4

Reporting Summary

## Data Availability

The authors declare that the data supporting the findings of this study are available within the paper and as Supplementary Material and Supplementary Methods files and may also be requested from M.M. Accession numbers for sequence data (fastq files) are provided in Supplementary Data 1.

## Code availability

R scripts and data for all THD models and figure generation can be found at https://github.com/rasigadelab/xdrtb_thd and at the Zenodo repository at https://zenodo.org/record/6826135 under 10.5281/zenodo.6826135.
