# Supplementary material for: Transcontinental spread and evolution of Mycobacterium tuberculosis W148 European/Russian clade toward extensively drug resistant tuberculosis
Source: Nat Commun. 2022 Aug 30;13:5105. doi: 10.1038/s41467-022-32455-1 (PMC9426364; doi:10.1038/s41467-022-32455-1)
Supplement: Supplementary file 1 — Supplementary Information [file 41467_2022_32455_MOESM1_ESM.pdf]

SUPPLEMENTARY NOTE

Evolving population, demographic models and clock calibration.

Before implementing Bayesian phylogenetic reconstructions and timing, we checked if our bacterial genomic dataset represents a measurably evolving population. For this purpose, we first measured genetic distances from a common ancestor against strain sampling time, which provided strong support for measurable accumulation of DNA sequence variation over time ($r^2 = 0.223$, $P < 0.001$) (Fig. 2a). The strength of the temporal signal was confirmed after applying tip-randomization (Fig. 2b). Moreover, the comparison of marginal likelihood scores of two alternative Bayesian models, i.e. fixed molecular clock and an uncorrelated relaxed clock clearly favored ($\log_{10}$ Bayes factor = 14.831) the relaxed molecular clock model. Furthermore, in order to accurately estimate the nucleotide substitution rate in a relaxed molecular clock model, different demographic models were compared (Table S1).

Table S1: Comparisons of different demographic and clock models under BEAST

Strains	Clock model	Demographic model	Substitution rate	TMRCA	Likelihood	BF (clock model)
731	Fixed	Constant	9.95×10^{-8}	245 (222-266)	- 44,182	
731	Tip dating, strict clock	Constant	1.02×10^{-7}	247 (214-281)	- 44,151	62
731	Tip dating, relaxed clock	Constant	1.14×10^{-7}	285 (183-402)	- 43,947	408
720	Tip dating, strict clock	Constant	1.05×10^{-7}	55 (47-65)	- 39,430	
720	Tip dating, relaxed clock	Constant	1.17×10^{-7}	68 (48-97)	- 39,215	430
720	Tip dating, strict clock	Skyline	9.11×10^{-8}	49 (39-59)	- 39,389	

Detailed THD model outputs and alternative models based on terminal branch lengths

As an alternative to THD success indices, we constructed models using the length of the terminal branches in the phylogeny in place of THD, as used elsewhere. Terminal branch lengths (TBLs) are interpreted as the delay between the last transmission event in the phylogeny and the sampling of the isolate. TBLs have been used previously as surrogate markers of transmission in M. tuberculosis.[1] TBL and THD appraoches differ as follows. While TBLs only consider the last transmission in an isolate's ancestry, THD considers all transmissions in a weighted fashion, such that older transmissions (deeper divergences in the phylogeny) are given less weight than recent transmission. The decrease of weight with transmission age is controlled by the timescale parameter (here, 10y). A small timescale puts more weight on recent transmission and, as the timescale decreases, the THD indices become increasingly correlated with (inverse) terminal branch lengths (Supplementary Figure 7).

Here, we provide detailed model outputs for all THD LMMs, as well as for their equivalent models using TBLs as the response variable. Correction for population structure in TBL-based models used the same procedure as THD-based models, although the selection procedure for genetic principal components resulted in a different set (PCs 2, 9, 11 and 18 for TBL-based models, vs. PCs 1, 2, 3 and 7 for THD-based models). Coefficients of the predictors of interest are highlighted.

1. Presence of compensatory mutations as a predictor of THD

```
Linear mixed model fit by REML. t-tests use Satterthwaite's method ['lmerModLmerTest']
Formula: thdw ~ mds1 + mds2 + mds3 + mds7 + possible_compensation + (1 |      country_of_isolation)
   Data: d

REML criterion at convergence: -5907.9

Scaled residuals: 
    Min      1Q  Median      3Q     Max 
-2.9740 -0.7335 -0.1280  0.6391  3.0962 

Random effects:
 Groups               Name        Variance  Std.Dev.
 country_of_isolation (Intercept) 2.069e-06 0.001438
 Residual                         1.356e-05 0.003683
Number of obs: 720, groups:  country_of_isolation, 14

Fixed effects:
                           Estimate Std. Error         df t value Pr(>|t|)    
(Intercept)               1.093e-02  5.726e-04  1.994e+01  19.088 2.79e-14 ***
mds1                      1.528e-04  2.160e-05  3.237e+02   7.075 9.29e-12 ***
mds2                      2.310e-04  2.871e-05  5.653e+02   8.044 5.14e-15 ***
mds3                     -1.849e-04  3.469e-05  4.042e+02  -5.330 1.64e-07 ***
mds7                     -2.205e-04  4.351e-05  7.075e+02  -5.068 5.13e-07 ***
possible_compensationyes -4.651e-04  3.930e-04  7.114e+02  -1.183    0.237    
---
Signif. codes:  0 '***' 0.001 '**' 0.01 '*' 0.05 '.' 0.1 ' ' 1

Correlation of Fixed Effects:
            (Intr) mds1   mds2   mds3   mds7  
mds1         0.292                            
mds2         0.205  0.315                     
mds3         0.057  0.396 -0.027              
mds7        -0.066 -0.108 -0.071 -0.039       
pssbl_cmpns -0.530 -0.234 -0.124  0.073  0.074
```

2. Presence of compensatory mutations as a predictor of terminal branch lengths

```
Linear mixed model fit by REML. t-tests use Satterthwaite's method ['lmerModLmerTest']
Formula: length ~ mds2 + mds11 + mds18 + mds9 + possible_compensation +      (1 | country_of_isolation)
   Data: d

REML criterion at convergence: -8394.1

Scaled residuals: 
    Min      1Q  Median      3Q     Max 
-2.1551 -0.6904 -0.2877  0.4933  4.2653 

Random effects:
 Groups               Name        Variance  Std.Dev. 
 country_of_isolation (Intercept) 7.962e-08 0.0002822
 Residual                         4.185e-07 0.0006469
Number of obs: 720, groups:  country_of_isolation, 14

Fixed effects:
                           Estimate Std. Error         df t value Pr(>|t|)    
(Intercept)               7.577e-04  1.027e-04  1.272e+01   7.376 6.11e-06 ***
mds2                     -2.785e-05  4.737e-06  6.351e+02  -5.879 6.68e-09 ***
mds11                     7.980e-05  1.109e-05  7.133e+02   7.198 1.55e-12 ***
mds18                     1.134e-04  1.528e-05  6.137e+02   7.423 3.83e-13 ***
mds9                      3.496e-05  9.362e-06  6.076e+02   3.734 0.000206 ***
possible_compensationyes -6.570e-05  6.659e-05  7.138e+02  -0.987 0.324152    
```

```
---
Signif. codes:  0 ‘***’ 0.001 ‘**’ 0.01 ‘*’ 0.05 ‘.’ 0.1 ‘ ’ 1

Correlation of Fixed Effects:
            (Intr) mds2   mds11  mds18  mds9
mds2         0.111
mds11        0.001  0.044
mds18        0.023  0.008  0.007
mds9         0.084  0.019  0.014  0.081
pssbl_cmpns -0.465 -0.019  0.005  0.020 -0.130
```

3. Resistance status as a predictor of THD

```
Linear mixed model fit by REML. t-tests use Satterthwaite's method ['lmerModLmerTest']
Formula: thdw ~ mds1 + mds2 + mds3 + mds7 + xdr2 + (1 | country_of_isolation)
   Data: d

REML criterion at convergence: -5488.3

Scaled residuals:
    Min      1Q  Median      3Q     Max
-2.8515 -0.7331 -0.1310  0.6237  3.1327

Random effects:
 Groups               Name        Variance  Std.Dev.
 country_of_isolation (Intercept) 2.234e-06 0.001494
 Residual                         1.351e-05 0.003675
Number of obs: 671, groups:  country_of_isolation, 14

Fixed effects:
              Estimate Std. Error         df t value Pr(>|t|)
(Intercept)  9.953e-03  5.780e-04  1.540e+01  17.221 1.77e-11 ***
mds1         1.263e-04  2.298e-05  3.000e+02   5.494 8.40e-08 ***
mds2         2.055e-04  2.942e-05  5.152e+02   6.986 8.80e-12 ***
mds3        -1.645e-04  3.648e-05  3.889e+02  -4.511 8.58e-06 ***
mds7        -2.300e-04  4.423e-05  6.595e+02  -5.200 2.66e-07 ***
xdr2pre-XDR  8.469e-04  3.859e-04  6.638e+02   2.194   0.0286 *
xdr2XDR      3.847e-04  4.999e-04  6.153e+02   0.770   0.4418
---
Signif. codes:  0 ‘***’ 0.001 ‘**’ 0.01 ‘*’ 0.05 ‘.’ 0.1 ‘ ’ 1

Correlation of Fixed Effects:
            (Intr) mds1   mds2   mds3   mds7   x2-XDR
mds1         0.303
mds2         0.201  0.325
mds3         0.073  0.348 -0.018
mds7        -0.059 -0.114 -0.082 -0.038
xdr2pre-XDR -0.453 -0.273 -0.114  0.034  0.005
xdr2XDR     -0.411 -0.236 -0.127  0.047  0.151  0.621

Type III Analysis of Variance Table with Satterthwaite's method
         Sum Sq    Mean Sq NumDF  DenDF F value    Pr(>F)
mds1 0.00040766 0.00040766     1 299.98 30.1831 8.399e-08 ***
mds2 0.00065910 0.00065910     1 515.17 48.7996 8.796e-12 ***
mds3 0.00027478 0.00027478     1 388.91 20.3447 8.576e-06 ***
mds7 0.00036522 0.00036522     1 659.53 27.0407 2.661e-07 ***
xdr2 0.00007274 0.00003637     2 647.96  2.6929   0.06844 .
---
Signif. codes:  0 ‘***’ 0.001 ‘**’ 0.01 ‘*’ 0.05 ‘.’ 0.1 ‘ ’ 1
```

4. Resistance status as a predictor of terminal branch lengths

```
Linear mixed model fit by REML. t-tests use Satterthwaite's method ['lmerModLmerTest']
Formula: length ~ mds2 + mds11 + mds18 + mds9 + xdr2 + (1 | country_of_isolation)
   Data: d

REML criterion at convergence: -7813.1

Scaled residuals:
    Min      1Q  Median      3Q     Max
-2.1812 -0.6326 -0.3108  0.5565  4.4975

Random effects:
 Groups               Name        Variance  Std.Dev.
 country_of_isolation (Intercept) 1.075e-07 0.0003278
 Residual                         4.075e-07 0.0006384
Number of obs: 671, groups:  country_of_isolation, 14
```

```
Fixed effects:
              Estimate Std. Error         df t value Pr(>|t|)    
(Intercept)  6.967e-04  1.114e-04  1.042e+01   6.257 7.87e-05 ***
mds2        -2.657e-05  4.825e-06  6.211e+02  -5.507 5.36e-08 ***
mds11        7.402e-05  1.122e-05  6.640e+02   6.596 8.63e-11 ***
mds18        1.166e-04  1.550e-05  5.982e+02   7.523 1.98e-13 ***
mds9         3.573e-05  9.661e-06  5.503e+02   3.699 0.000239 ***
xdr2pre-XDR  3.112e-06  6.433e-05  6.638e+02   0.048 0.961438    
xdr2XDR     -7.363e-05  8.379e-05  6.375e+02  -0.879 0.379818    
---
Signif. codes:  0 ‘***’ 0.001 ‘**’ 0.01 ‘*’ 0.05 ‘.’ 0.1 ‘ ’ 1

Correlation of Fixed Effects:
            (Intr) mds2   mds11  mds18  mds9   x2-XDR
mds2         0.103                                   
mds11        0.016  0.053                            
mds18       -0.001  0.003  0.017                     
mds9         0.021  0.028  0.030  0.105              
xdr2pre-XDR -0.346 -0.002  0.004  0.092  0.001       
xdr2XDR     -0.317 -0.025 -0.015  0.072 -0.028  0.600

Type III Analysis of Variance Table with Satterthwaite's method
         Sum Sq    Mean Sq NumDF  DenDF F value    Pr(>F)    
mds2  1.2358e-05 1.2358e-05     1 621.12 30.3233 5.358e-08 ***
mds11 1.7731e-05 1.7731e-05     1 664.00 43.5078 8.627e-11 ***
mds18 2.3063e-05 2.3063e-05     1 598.16 56.5899 1.979e-13 ***
mds9  5.5749e-06 5.5749e-06     1 550.29 13.6793 0.0002386 ***
xdr2  5.2550e-07 2.6280e-07     2 655.09  0.6448 0.5251177    
---
Signif. codes:  0 ‘***’ 0.001 ‘**’ 0.01 ‘*’ 0.05 ‘.’ 0.1 ‘ ’ 1
```

5. Resistance status, compensatory mutation and their interaction as predictors of THD

```
Linear mixed model fit by REML. t-tests use Satterthwaite's method ['lmerModLmerTest']
Formula: thdw ~ mds1 + mds2 + mds3 + mds7 + possible_compensation * xdr2 +      (1 | country_of_isolation)
   Data: d[]

REML criterion at convergence: -5460.6

Scaled residuals: 
    Min      1Q  Median      3Q     Max 
-2.8438 -0.7148 -0.1210  0.5873  3.1090 

Random effects:
 Groups               Name        Variance  Std.Dev.
 country_of_isolation (Intercept) 1.877e-06 0.001370
 Residual                         1.341e-05 0.003662
Number of obs: 671, groups:  country_of_isolation, 14

Fixed effects:
                                       Estimate Std. Error         df t value Pr(>|t|)    
(Intercept)                           1.097e-02  7.510e-04  5.314e+01  14.601  < 2e-16 ***
mds1                                  1.172e-04  2.340e-05  2.424e+02   5.007 1.06e-06 ***
mds2                                  1.960e-04  2.963e-05  5.159e+02   6.614 9.38e-11 ***
mds3                                 -1.618e-04  3.620e-05  3.516e+02  -4.469 1.06e-05 ***
mds7                                 -2.254e-04  4.421e-05  6.580e+02  -5.100 4.45e-07 ***
possible_compensationyes             -1.214e-03  6.378e-04  6.572e+02  -1.903  0.05748 .  
xdr2pre-XDR                          -6.101e-04  8.358e-04  6.582e+02  -0.730  0.46563    
xdr2XDR                              -3.650e-03  1.599e-03  2.289e+02  -2.283  0.02336 *  
possible_compensationyes:xdr2pre-XDR  1.904e-03  9.428e-04  6.610e+02   2.019  0.04388 *  
possible_compensationyes:xdr2XDR      4.568e-03  1.666e-03  2.753e+02   2.742  0.00651 ** 
---
Signif. codes:  0 ‘***’ 0.001 ‘**’ 0.01 ‘*’ 0.05 ‘.’ 0.1 ‘ ’ 1

Correlation of Fixed Effects:
            (Intr) mds1   mds2   mds3   mds7   pssbl_ x2-XDR xd2XDR p_:2-X
mds1         0.241                                                        
mds2         0.177  0.350                                                 
mds3         0.038  0.315 -0.023                                          
mds7        -0.066 -0.125 -0.094 -0.034                                   
pssbl_cmpns -0.679 -0.020 -0.041  0.017  0.032                            
xdr2pre-XDR -0.533 -0.001 -0.007 -0.025 -0.001  0.592                     
xdr2XDR     -0.332  0.043  0.067  0.042 -0.004  0.310  0.247              
pssb_:2-XDR  0.423 -0.140 -0.055  0.046  0.006 -0.670 -0.888 -0.210       
pssbl_:2XDR  0.276 -0.128 -0.114 -0.023  0.052 -0.374 -0.237 -0.947  0.299
```

6. Resistance status, compensatory mutation and their interaction as predictors of terminal branch lengths

```
Linear mixed model fit by REML. t-tests use Satterthwaite's method ['lmerModLmerTest']
```

```
Formula: length ~ mds2 + mds11 + mds18 + mds9 + possible_compensation *      xdr2 + (1 | country_of_isolation)
   Data: d[]

REML criterion at convergence: -7773.8

Scaled residuals:
    Min      1Q  Median      3Q     Max
-2.1840 -0.6374 -0.2850  0.5831  4.5221

Random effects:
 Groups               Name        Variance  Std.Dev.
 country_of_isolation (Intercept) 9.261e-08 0.0003043
 Residual                         4.054e-07 0.0006367
Number of obs: 671, groups:  country_of_isolation, 14

Fixed effects:
                                       Estimate Std. Error         df t value Pr(>|t|)
(Intercept)                           6.712e-04  1.383e-04  2.842e+01   4.854 3.99e-05 ***
mds2                                 -2.530e-05  4.824e-06  6.222e+02  -5.243 2.17e-07 ***
mds11                                 7.925e-05  1.134e-05  6.600e+02   6.987 6.90e-12 ***
mds18                                 1.114e-04  1.557e-05  5.006e+02   7.152 3.06e-12 ***
mds9                                  4.258e-05  9.941e-06  6.534e+02   4.284 2.11e-05 ***
possible_compensationyes              1.340e-06  1.113e-04  6.552e+02   0.012  0.99040
xdr2pre-XDR                          -2.357e-05  1.457e-04  6.552e+02  -0.162  0.87148
xdr2XDR                               6.911e-04  2.990e-04  2.556e+02   2.311  0.02163 *
possible_compensationyes:xdr2pre-XDR  2.610e-05  1.626e-04  6.606e+02   0.160  0.87254
possible_compensationyes:xdr2XDR     -8.049e-04  3.103e-04  2.987e+02  -2.594  0.00995 **
---
Signif. codes:  0 ‘***’ 0.001 ‘**’ 0.01 ‘*’ 0.05 ‘.’ 0.1 ‘ ’ 1

Correlation of Fixed Effects:
            (Intr) mds2   mds11  mds18  mds9   pssbl_ x2-XDR xd2XDR p_:2-X
mds2         0.093
mds11       -0.018  0.065
mds18        0.048 -0.009 -0.008
mds9         0.023  0.050  0.068  0.067
pssbl_cmpns -0.640 -0.031  0.029 -0.064 -0.040
xdr2pre-XDR -0.509 -0.010  0.023 -0.026 -0.038  0.595
xdr2XDR     -0.318  0.070  0.156 -0.107  0.217  0.291  0.229
pssb_:2-XDR  0.447  0.009 -0.028  0.077  0.039 -0.687 -0.897 -0.198
pssbl_:2XDR  0.292 -0.077 -0.165  0.136 -0.225 -0.358 -0.221 -0.958  0.271
```

7. No. of resistance mutations as a predictor of THD

```
Linear mixed model fit by REML. t-tests use Satterthwaite's method ['lmerModLmerTest']
Formula: thdw ~ mds1 + mds2 + mds3 + mds7 + genotypic_resistances + (1 |      country_of_isolation)
   Data: d

REML criterion at convergence: -5918.2

Scaled residuals:
    Min      1Q  Median      3Q     Max
-2.9084 -0.7484 -0.1329  0.5886  3.2120

Random effects:
 Groups               Name        Variance  Std.Dev.
 country_of_isolation (Intercept) 1.225e-06 0.001107
 Residual                         1.338e-05 0.003658
Number of obs: 720, groups:  country_of_isolation, 14

Fixed effects:
                        Estimate Std. Error         df t value Pr(>|t|)
(Intercept)            1.261e-02  6.412e-04  4.003e+01  19.659  < 2e-16 ***
mds1                   1.529e-04  2.021e-05  1.492e+02   7.568 3.62e-12 ***
mds2                   2.149e-04  2.791e-05  3.642e+02   7.701 1.28e-13 ***
mds3                  -1.916e-04  3.382e-05  2.514e+02  -5.666 3.99e-08 ***
mds7                  -2.335e-04  4.327e-05  7.098e+02  -5.397 9.25e-08 ***
genotypic_resistances -3.161e-04  7.942e-05  6.487e+02  -3.980 7.66e-05 ***
---
Signif. codes:  0 ‘***’ 0.001 ‘**’ 0.01 ‘*’ 0.05 ‘.’ 0.1 ‘ ’ 1

Correlation of Fixed Effects:
            (Intr) mds1   mds2   mds3   mds7
mds1         0.167
mds2         0.045  0.289
mds3        -0.036  0.392 -0.001
mds7        -0.096 -0.090 -0.050 -0.025
gntypc_rsst -0.779 -0.031  0.082  0.149  0.096
```

8. No. of resistance mutations as a predictor of terminal branch length

```
Linear mixed model fit by REML. t-tests use Satterthwaite's method ['lmerModLmerTest']
Formula: length ~ mds2 + mds11 + mds18 + mds9 + genotypic_resistances +      (1 | country_of_isolation)
   Data: d

REML criterion at convergence: -8390.1

Scaled residuals: 
    Min      1Q  Median      3Q     Max 
-2.1758 -0.6936 -0.2897  0.4888  4.2595 

Random effects:
 Groups               Name        Variance  Std.Dev. 
 country_of_isolation (Intercept) 8.260e-08 0.0002874
 Residual                         4.188e-07 0.0006472
Number of obs: 720, groups:  country_of_isolation, 14

Fixed effects:
                        Estimate Std. Error         df t value Pr(>|t|)    
(Intercept)            7.391e-04  1.284e-04  2.569e+01   5.756 4.84e-06 ***
mds2                  -2.815e-05  4.787e-06  6.228e+02  -5.881 6.67e-09 ***
mds11                  7.973e-05  1.110e-05  7.135e+02   7.183 1.72e-12 ***
mds18                  1.137e-04  1.529e-05  6.208e+02   7.433 3.54e-13 ***
mds9                   3.424e-05  9.390e-06  5.755e+02   3.646  0.00029 ***
genotypic_resistances -4.559e-06  1.412e-05  6.659e+02  -0.323  0.74693    
---
Signif. codes:  0 ‘***’ 0.001 ‘**’ 0.01 ‘*’ 0.05 ‘.’ 0.1 ‘ ’ 1

Correlation of Fixed Effects:
            (Intr) mds2   mds11  mds18  mds9  
mds2        -0.014                            
mds11       -0.020  0.049                     
mds18        0.015  0.011  0.008              
mds9         0.118 -0.002  0.011  0.083       
gntypc_rsst -0.696  0.138  0.034  0.017 -0.142
```

9. Three-way interaction model of THD

```
Linear mixed model fit by REML. t-tests use Satterthwaite's method ['lmerModLmerTest']
Formula: thdw ~ mds1 + mds2 + mds3 + mds7 + genotypic_resistances:possible_compensation:xdr2 +      (1 | 
country_of_isolation)
   Data: d[]

REML criterion at convergence: -5468.3

Scaled residuals: 
    Min      1Q  Median      3Q     Max 
-2.8405 -0.7066 -0.1141  0.5880  3.0909 

Random effects:
 Groups               Name        Variance  Std.Dev.
 country_of_isolation (Intercept) 1.493e-06 0.001222
 Residual                         1.262e-05 0.003553
Number of obs: 671, groups:  country_of_isolation, 14

Fixed effects:
                                                            Estimate Std. Error         df t value Pr(>|t|)    
(Intercept)                                                1.629e-02  9.544e-04  1.440e+02  17.065  < 2e-16 ***
mds1                                                       7.996e-05  2.285e-05  2.355e+02   3.499 0.000558 ***
mds2                                                       1.438e-04  2.964e-05  4.815e+02   4.851 1.66e-06 ***
mds3                                                      -1.859e-04  3.505e-05  3.242e+02  -5.303 2.11e-07 ***
mds7                                                      -2.231e-04  4.265e-05  6.575e+02  -5.231 2.27e-07 ***
genotypic_resistances:possible_compensationno:xdr2MDR     -1.221e-03  2.311e-04  6.585e+02  -5.283 1.73e-07 ***
genotypic_resistances:possible_compensationyes:xdr2MDR    -1.375e-03  1.907e-04  6.593e+02  -7.213 1.51e-12 ***
genotypic_resistances:possible_compensationno:xdr2pre-XDR -8.567e-04  1.498e-04  6.599e+02  -5.720 1.62e-08 ***
genotypic_resistances:possible_compensationyes:xdr2pre-XDR -8.006e-04  1.312e-04  6.599e+02  -6.102 1.79e-09 ***
genotypic_resistances:possible_compensationno:xdr2XDR     -1.167e-03  2.098e-04  3.216e+02  -5.565 5.53e-08 ***
genotypic_resistances:possible_compensationyes:xdr2XDR    -6.488e-04  1.047e-04  6.565e+02  -6.199 1.00e-09 ***
```

10. Three-way interaction model of terminal branch length

```
Linear mixed model fit by REML. t-tests use Satterthwaite's method ['lmerModLmerTest']
Formula: length ~ mds2 + mds11 + mds18 + mds9 + genotypic_resistances:possible_compensation:xdr2 +      (1 | 
country_of_isolation)
   Data: d[]
```

```
REML criterion at convergence: -7738.8

Scaled residuals: 
    Min      1Q  Median      3Q     Max 
-2.3424 -0.6362 -0.3092  0.5745  4.5399 

Random effects:
 Groups               Name        Variance  Std.Dev. 
 country_of_isolation (Intercept) 9.392e-08 0.0003065
 Residual                         4.040e-07 0.0006356
Number of obs: 671, groups:  country_of_isolation, 14

Fixed effects:
                                                             Estimate Std. Error         df t value Pr(>|t|)    
(Intercept)                                                 4.290e-04  1.830e-04  7.009e+01   2.345  0.02188 *  
mds2                                                       -2.369e-05  4.921e-06  6.212e+02  -4.814 1.86e-06 ***
mds11                                                       8.034e-05  1.133e-05  6.592e+02   7.089 3.49e-12 ***
mds18                                                       1.095e-04  1.560e-05  5.026e+02   7.019 7.28e-12 ***
mds9                                                        4.072e-05  1.001e-05  6.490e+02   4.067 5.34e-05 ***
genotypic_resistances:possible_compensationno:xdr2MDR       5.390e-05  3.989e-05  6.600e+02   1.351  0.17709    
genotypic_resistances:possible_compensationyes:xdr2MDR      5.098e-05  3.290e-05  6.559e+02   1.549  0.12176    
genotypic_resistances:possible_compensationno:xdr2pre-XDR   3.274e-05  2.602e-05  6.597e+02   1.258  0.20877    
genotypic_resistances:possible_compensationyes:xdr2pre-XDR  3.667e-05  2.324e-05  6.577e+02   1.578  0.11496    
genotypic_resistances:possible_compensationno:xdr2XDR       1.144e-04  3.896e-05  3.128e+02   2.936  0.00357 ** 
genotypic_resistances:possible_compensationyes:xdr2XDR      1.227e-05  1.866e-05  6.453e+02   0.657  0.51118    
```

≥

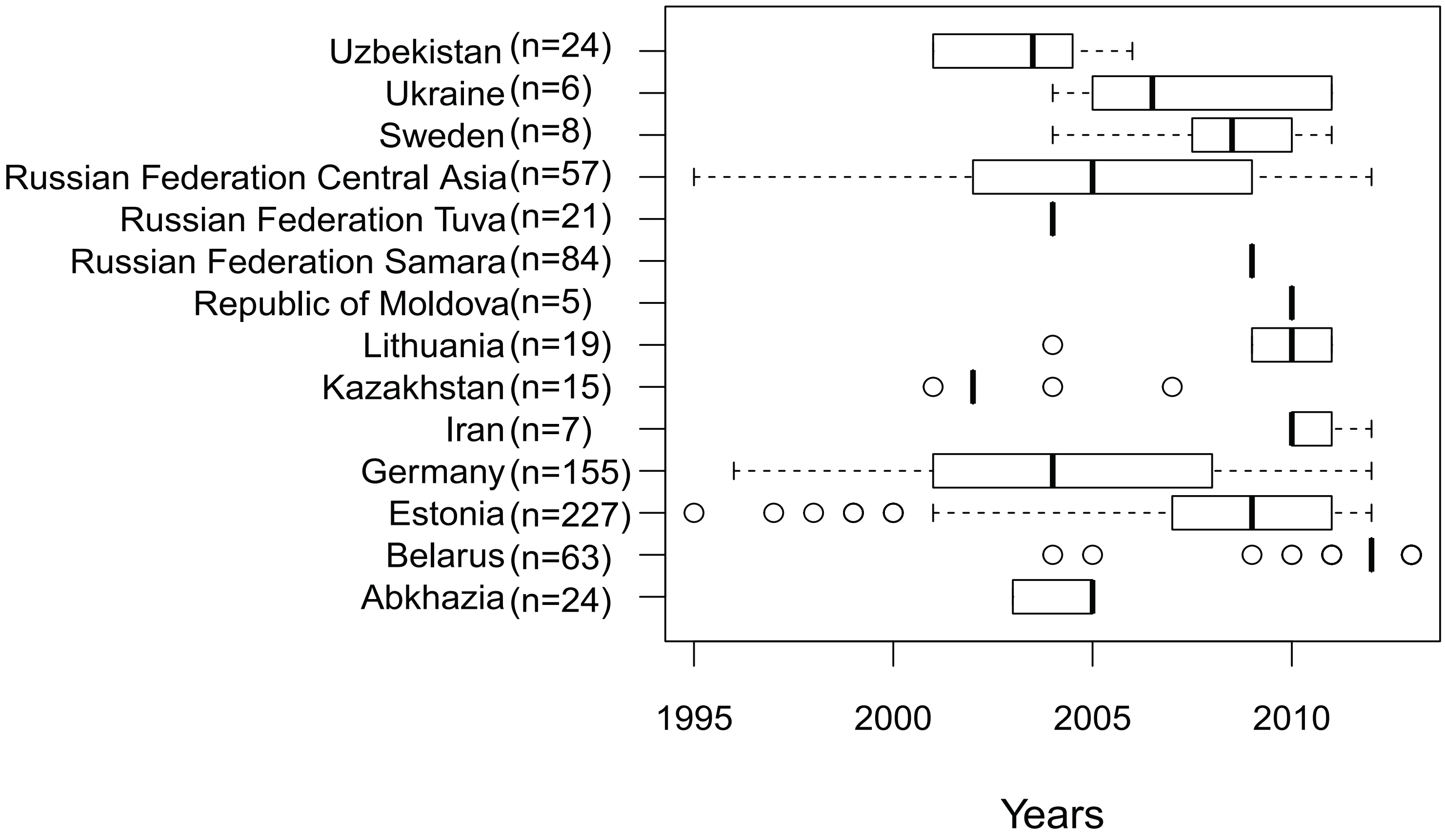

Supplementary Figure 1
Box-plots of the sampling years according to the regional boundaries. Solid bars indicate the median, boxes represent the inter quartile range (IQR), whiskers extend to 1.5x IQR. Outliers are indicated by individual dots.

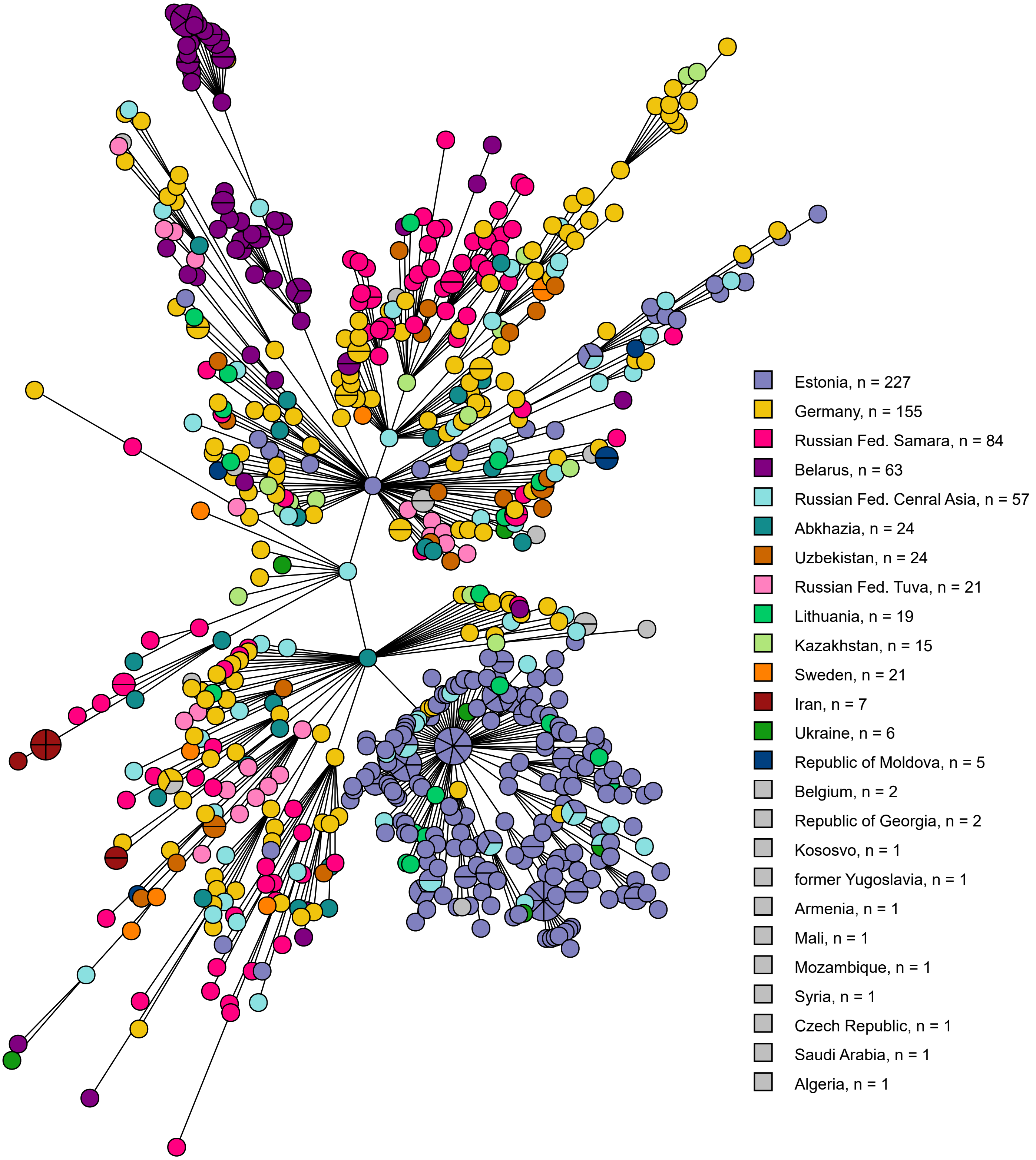

Supplementary Figure 2
Minimum spanning tree based on SNPs of the 720 W148 strains. Strains are colorized according to their location. Strains represented in grey correspond to locations with small samples sizes (N<5), not represented on Fig.1a. The length of the links is proportional to the number of SNPs between strains.

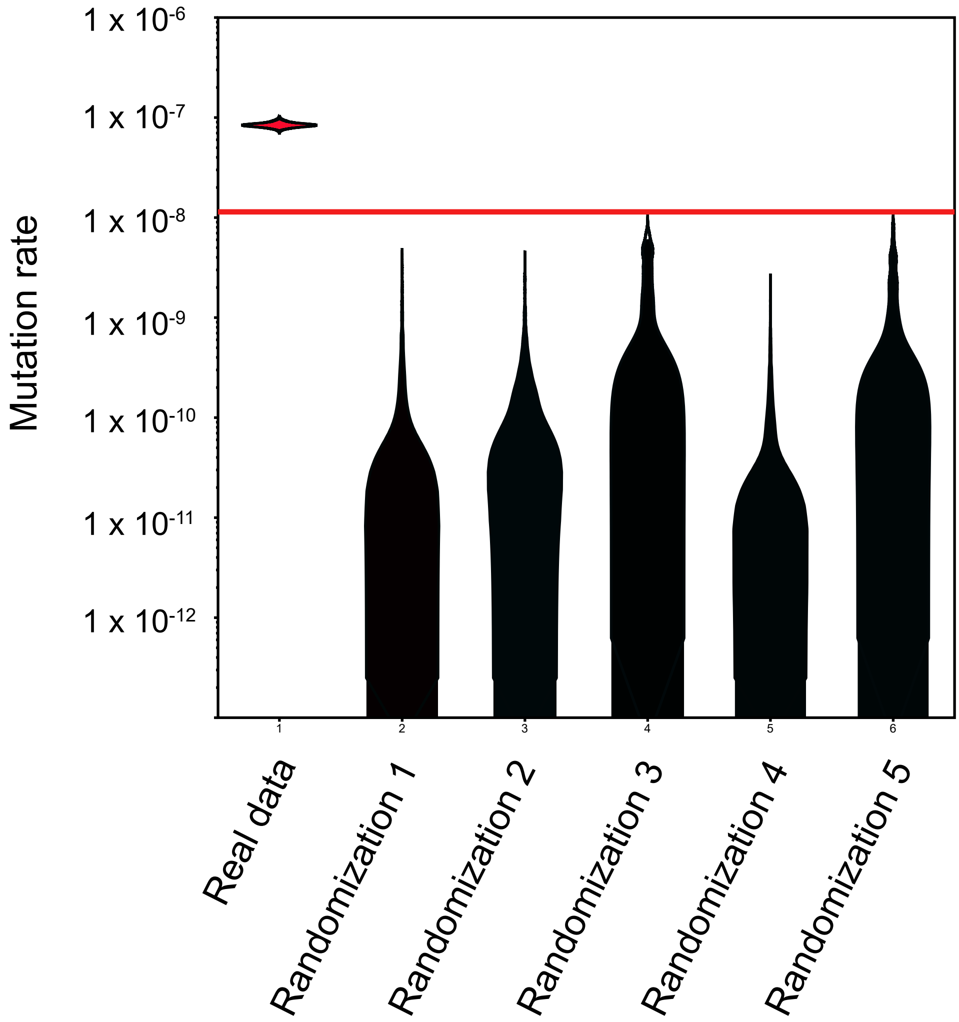

Supplementary Figure 3
Tip randomization test on molecular clock rates under a Bayesian skyline model and a strict clock. Calculated clock rates are represented using non-parametric violin plots that contain all data points. Note that there is no overlap between rates calculated with randomized datasets and the real dataset.

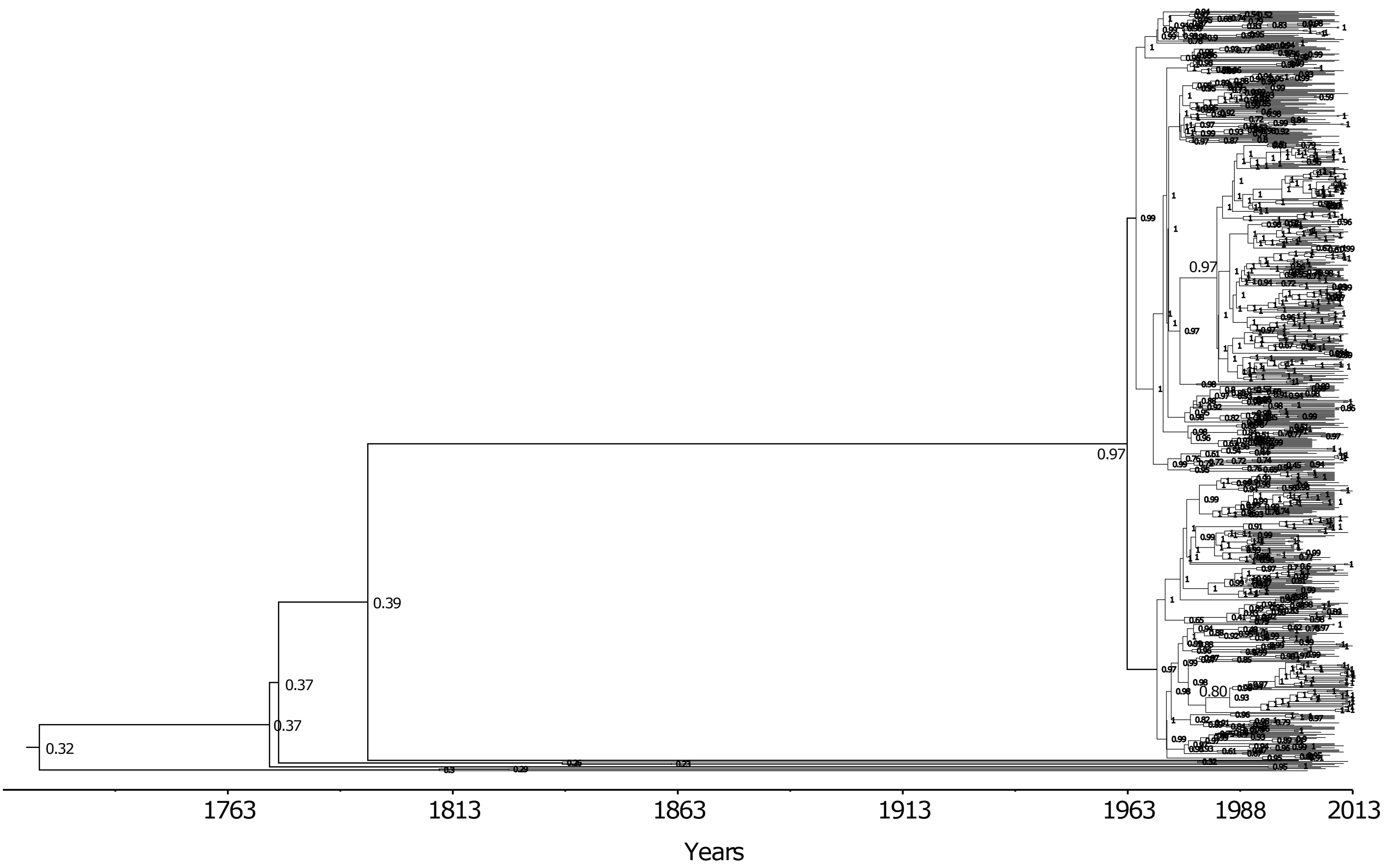

Supplementary Figure 4

W148 Bayesian maximum clade credibility phylogeny inferred under a skyline model using a HKY substitution model. Implementing a tip dating approach resulted in an estimated substitution rate of 1.12 x 10-7 substitutions per nucleotide per year. Note that the W148 TMRCA is in the early 1960’s.

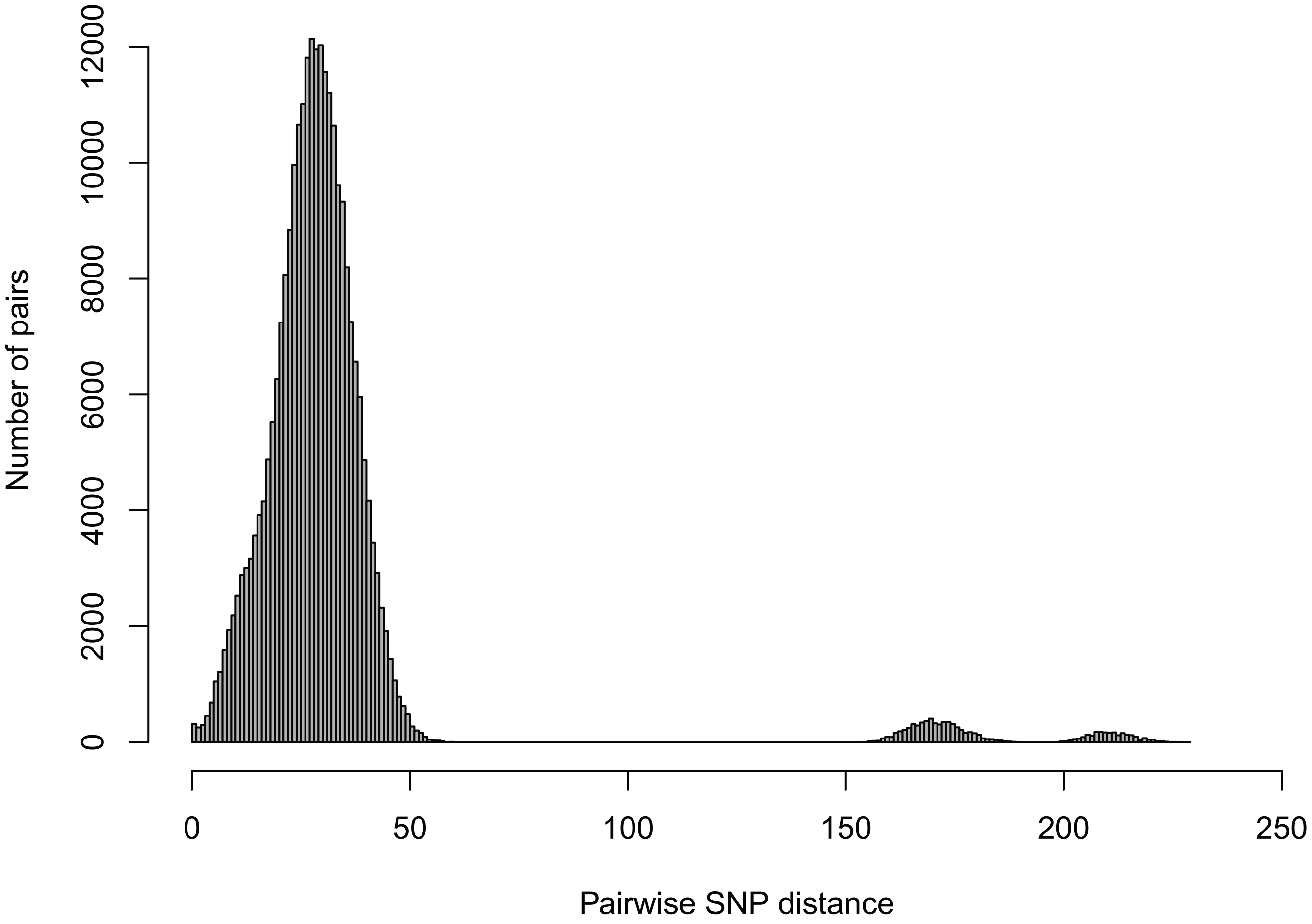

Supplementary Figure 5
Pairwise single nucleotide polymorphism (SNP) distances of the 731 M. tuberculosis strains.

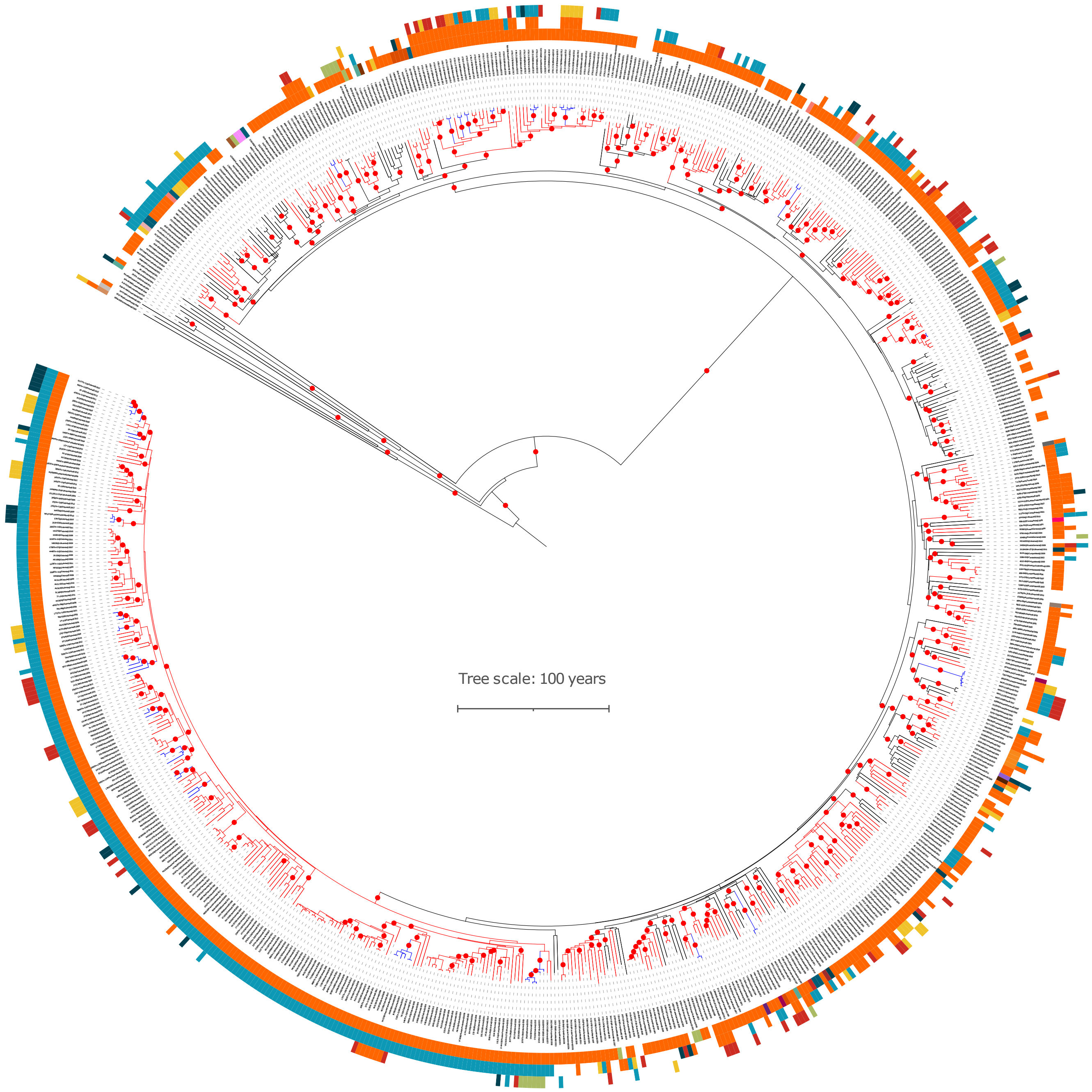

Supplementary Figure 6

W148 Bayesian maximum clade credibility (MCC) phylogeny. Phylogeny inferred under a skyline model using a HKY substitution model for 720 W148 isolates and 11 lineage 2 outgroup isolates. Nodes with a posterior probability >0.8 are indicated by red dots. Clades with a red branch color represent multidrug resistant (MDR) clades (i.e. at least isoniazid and rifampicin resistance), clades with a blue branch color represent MDR clades with additional resistance against a fluoroquinolone and a second-line injectable drug (i.e. extensively drug resistance (XDR) according to WHO classification until the end of 2020). The time from the most recent common ancestor (MRCA) of a red MDR clade to the descending MRCA of a blue XDR clade was averaged for 25 of such events (MDR to XDR). Color bars at the outer circle represent genotypic drug resistance determinants, inner to outer ring: rifampicin resistance, second-line injectable drug resistance, fluoroquinolone resistance. Different colors indicate different mutations. The MCC tree can be explored in detail via https://itol.embl.de/tree/1953756242471111547031648.

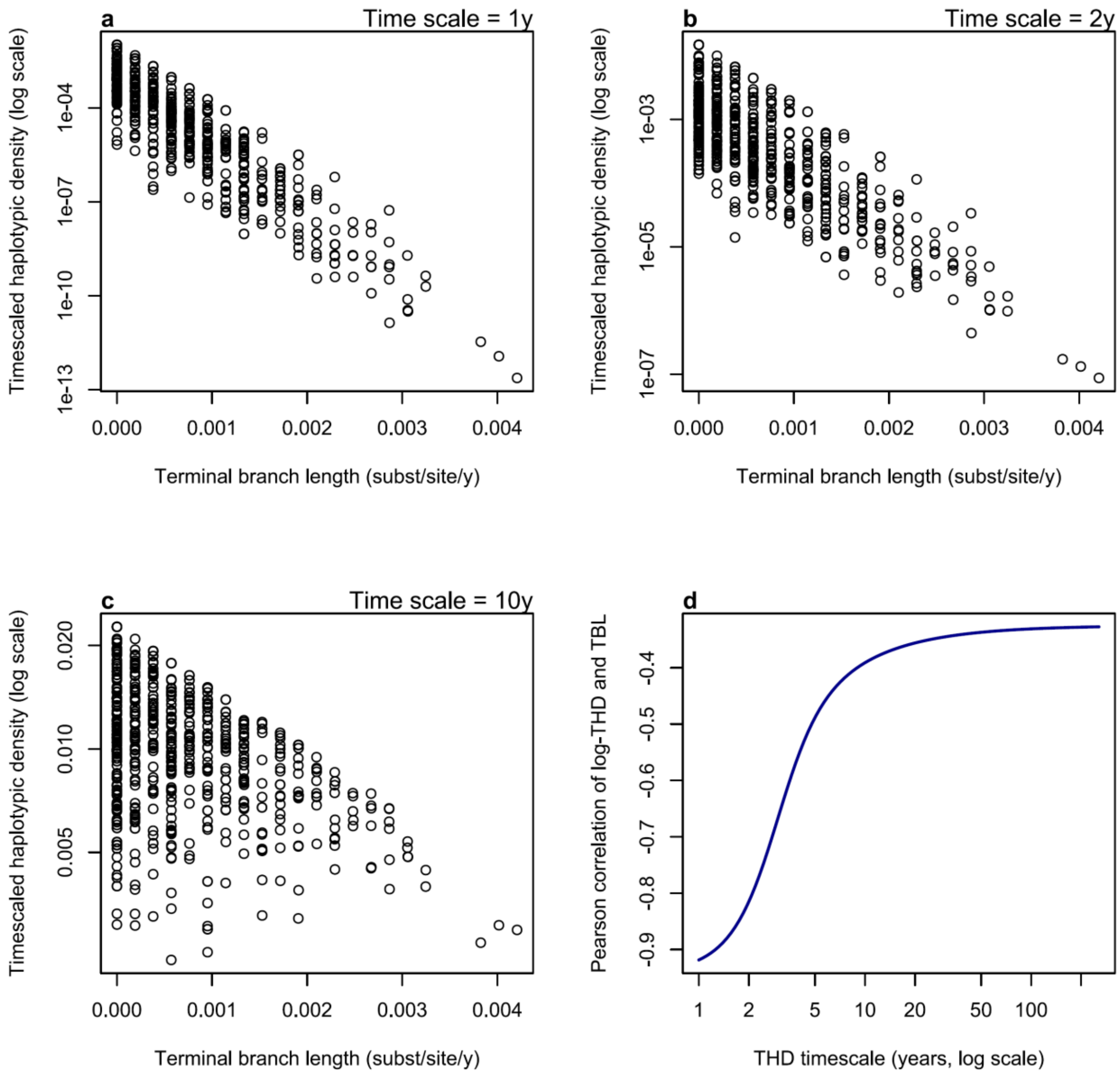

Supplementary Figure 7. Pearson correlation of timescaled haplotypic densities (THDs) and terminal branch lengths (TBLs) as a function of the THD time scale. The negative correlation of log-THD and TBL was strongest with a very small timescale of 1y (a) and decreased as the time scale increased to 2y (b) or 10y (c; as used in the rest of the analyses). Overall, the correlation strength decreases as the time scale increases and as information from older divergence events (which are ignored by TBL) is given more weight (d). This suggests that TBLs can be roughly interpreted as very short-term THD indices.

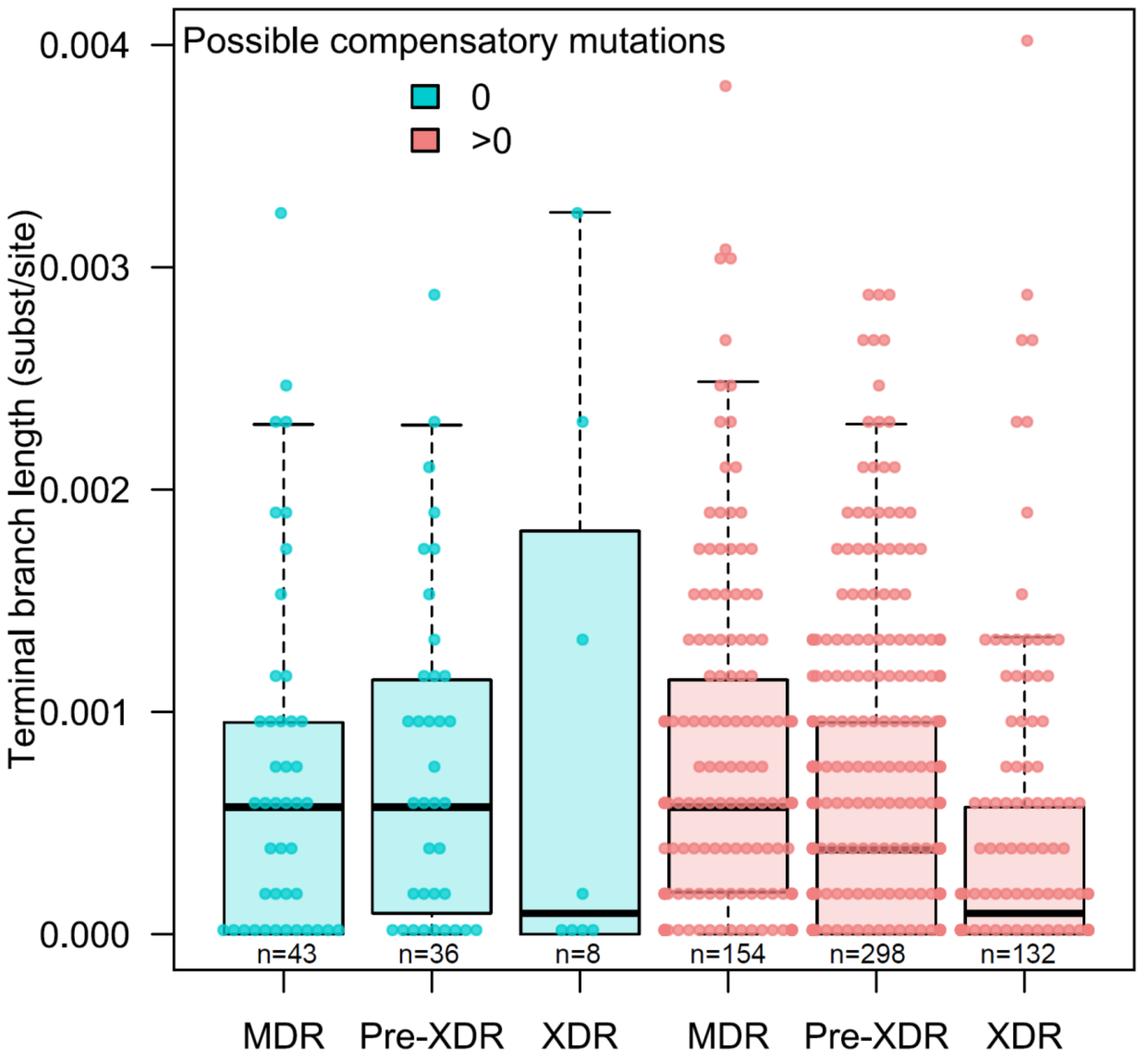

Supplementary Figure 8. Evolution of terminal branch lengths with resistance status (MDR, and pre-XDR, XDR according to definitions in place in 2020) in isolates with and without possible compensatory mutations. The median terminal branch length decreased with resistance status both in isolates lacking compensatory mutations and in isolates harboring these mutations. Solid bars indicate the median, boxes represent the inter quartile range (IQR), whiskers extend to 1.5x IQR.

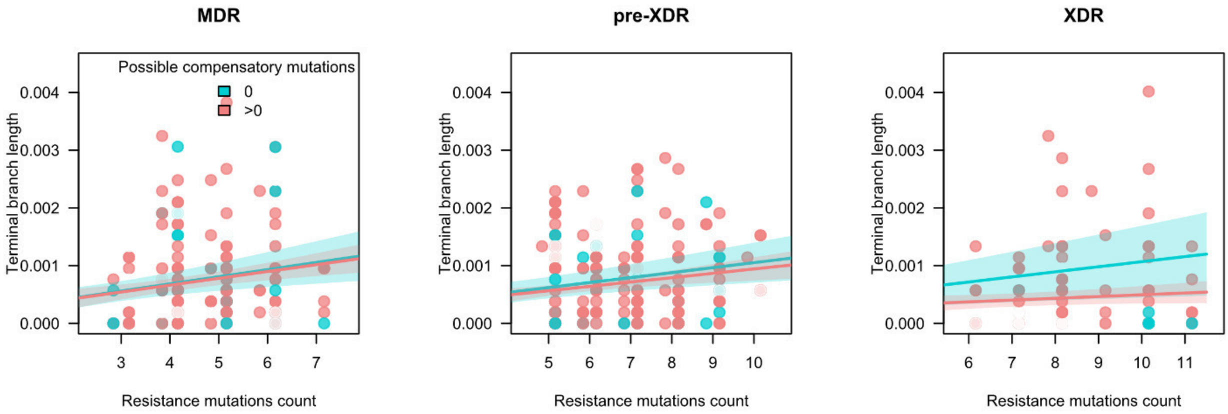

Supplementary Figure 9. Terminal branch lengths increase with the accumulation of resistance-conferring mutations within MDR, pre-XDR and XDR isolates. Colored lines and bands denote terminal branch lengths (point estimate and 95% confidence interval, respectively) predicted by a linear mixed model adjusted for population structure.

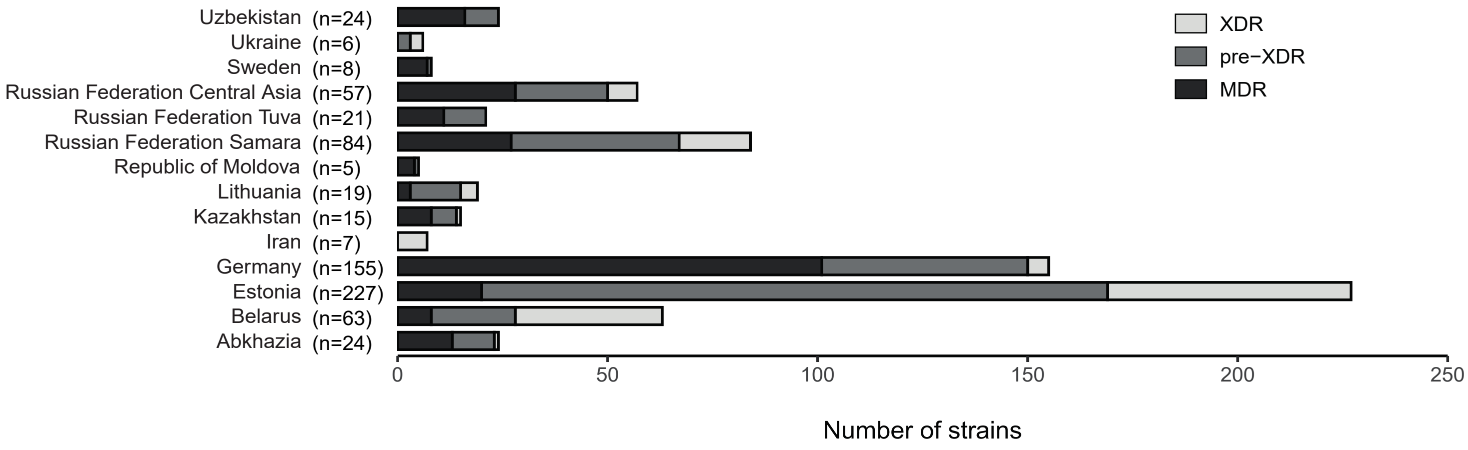

Supplementary Figure 10

Regional dependent antibiotic resistance profiles of the sample collection. MDR=multidrug resistant, (pre-)XDR= extensively drug resistant
