# Supplementary material for: Transcontinental spread and evolution of Mycobacterium tuberculosis W148 European/Russian clade toward extensively drug resistant tuberculosis
Source: Nat Commun. 2022 Aug 30;13:5105. doi: 10.1038/s41467-022-32455-1 (PMC9426364; doi:10.1038/s41467-022-32455-1)
Supplement: Supplementary file 2 — Description of Additional Supplementary Files [file 41467_2022_32455_MOESM2_ESM.pdf]

Description of Additional Supplementary Files

File Name: Supplementary Data 1
Description: Overview of the 731 strains (including 720 Russian clone/W148 strains) implemented for the whole-genome Bayesian inferences and phylogenetic reconstructions.

File Name: Supplementary Data 2
Description: List of the W148 specific SNP's under potential positive selection according to HOMOPLASYFINDER.

File Name: Supplementary Data 3
Description: Sampling rationale, geographic origin and estimated fractions of the studied samples.

File Name: Supplementary Data 4
Description: Resistance target genes and rationales for molecular drug resistance prediction.
