# Supplementary material for: Transcontinental spread and evolution of Mycobacterium tuberculosis W148 European/Russian clade toward extensively drug resistant tuberculosis
Source: Nat Commun. 2022 Aug 30;13:5105. doi: 10.1038/s41467-022-32455-1 (PMC9426364; doi:10.1038/s41467-022-32455-1)
Supplement: Supplementary file 7 — Reporting Summary [file 41467_2022_32455_MOESM7_ESM.pdf]

nature research

Corresponding author(s): Prof. Thierry Wirth

Last updated by author(s): Jul 14, 2022

Reporting Summary

Nature Research wishes to improve the reproducibility of the work that we publish. This form provides structure for consistency and transparency in reporting. For further information on Nature Research policies, see our Editorial Policies and the Editorial Policy Checklist.

Statistics

For all statistical analyses, confirm that the following items are present in the figure legend, table legend, main text, or Methods section.

n/a	Confirmed	
☐	☒	The exact sample size (n) for each experimental group/condition, given as a discrete number and unit of measurement
☒	☐	A statement on whether measurements were taken from distinct samples or whether the same sample was measured repeatedly
☐	☒	The statistical test(s) used AND whether they are one- or two-sided<br>*Only common tests should be described solely by name; describe more complex techniques in the Methods section.*
☐	☒	A description of all covariates tested
☐	☒	A description of any assumptions or corrections, such as tests of normality and adjustment for multiple comparisons
☐	☒	A full description of the statistical parameters including central tendency (e.g. means) or other basic estimates (e.g. regression coefficient) AND variation (e.g. standard deviation) or associated estimates of uncertainty (e.g. confidence intervals)
☐	☒	For null hypothesis testing, the test statistic (e.g. F, t, r) with confidence intervals, effect sizes, degrees of freedom and P value noted<br>*Give P values as exact values whenever suitable.*
☐	☒	For Bayesian analysis, information on the choice of priors and Markov chain Monte Carlo settings
☒	☐	For hierarchical and complex designs, identification of the appropriate level for tests and full reporting of outcomes
☐	☒	Estimates of effect sizes (e.g. Cohen's d, Pearson's r), indicating how they were calculated

Our web collection on statistics for biologists contains articles on many of the points above.

Software and code

Policy information about availability of computer code

Data collection	We sequenced 537 isolates for this study, and supplemented this cohort with 194 published datasets from SRA/ENA. Rationales and selection critieria are detailed under "sample size" below, and in the method section of the manuscript. Fastq files/reads were mapped to the M. tuberculosis H37Rv genome (GenBank ID: NC_000962.3) and dataset were concatenated with MTBseq v1.0.4. The resulting concatenated sequence alignment was used for further phylogenetic and statistical analysis.
Data analysis	Maximum-likelihood (ML) approach with PHYML 3.412, ML-tree midpoint rooting with FIGTREE, linear regression of root-to-tip distances against sampling time was performed using TEMPEST1.5, to assess robustness of our root-to-tip regression we used LMPERM Package, date randomization was performed with tipdating beast (R package), bayesian coalescent analysis with BEAST v2.3.2 and evaluated with TRACER V1.6., nucleotide diversity pi per regions analyzed with the R packages APE and PEGAS, minimum spanning tree was produced using BIONUMERICS version 7.6., linear mixed models (LMMs) using R package LME, significance of LMM coefficients was assessed using R package LMERTEST, R software version 4.0.2, homoplasy was analyzed with HomoplasyFinder (accessed at 26.10.2020), linkage of ppe38 gene locus with an IS6110 element was identified by bowtie 2 (for the last analysis reads were mapped with CLC Genomic Workbench v22.0)

For manuscripts utilizing custom algorithms or software that are central to the research but not yet described in published literature, software must be made available to editors and reviewers. We strongly encourage code deposition in a community repository (e.g. GitHub). See the Nature Research guidelines for submitting code & software for further information.

Data

Policy information about availability of data

All manuscripts must include a data availability statement. This statement should provide the following information, where applicable:

- Accession codes, unique identifiers, or web links for publicly available datasets
- A list of figures that have associated raw data
- A description of any restrictions on data availability

The authors declare that the data supporting the findings of this study are available within the paper and as Supplementary Material and Supplementary Methods files and may also be requested from M.M. Accession numbers are given in Table S1.

Field-specific reporting

Please select the one below that is the best fit for your research. If you are not sure, read the appropriate sections before making your selection.

☒ Life sciences ☐ Behavioural & social sciences ☐ Ecological, evolutionary & environmental sciences

For a reference copy of the document with all sections, see nature.com/documents/nr-reporting-summary-flat.pdf

Life sciences study design

All studies must disclose on these points even when the disclosure is negative.

Sample size	Overall, 731 Mtbc isolates were identified based on characteristic Mtbc genotyping patterns classifying them as W148 Beijing family (i.e. that includes mycobacterial interspersed repetitive units (MIRU) genotype '100-32', and a characteristic IS6110 restriction fragment length polymorphism (RFLP) banding pattern described previously. Of those, 720 isolates had specific genetic polymorphisms identifying them as W148 Europe/Russian outbreak clade. Isolates were sampled in 23 different countries between 1995-2013 under different study rationales. The majority of the data is part of a broader international genotyping project that was funded until 2013, and we retrospectively leveraged the unique selection of W148 strains for whole genome sequencing, and supplemented this cohort with publicly available lineage 2 W148 genomes. A detailed overview of the sampling scheme per country and estimates for the regional prevalence of W148 strains are given in the supplement (Table S4). The global dataset entails 537 newly sequenced genomes, plus another 194 publicly available datasets. Ethical approval was granted by the ethic commission of the University of Lübeck, Germany.
Data exclusions	No data excluded
Replication	Maximum likelihood phylogenetic frameworks, bayesian coalescent analysis, linear and logistic regressions were performed with permutations/pseudoreplicates as detailed in the method section for each test. All replications supported the results in this manuscript.
Randomization	Isolates were allocated to groups based on their genotypic drug resistance profile. This allocation was not random. We stratified isolates to multidrug resistant (MDR), extensively drug resistant (XDR), and pre-XDR, where pre-XDR indicates MDR (i.e. resistance to at least isoniazid and rifampicin) with additional resistance to a second-line injectable drug or a fluoroquinolone, and XDR includes both, resistances against a second-line injectable drug and a fluoroquinolone in an MDR background.
Blinding	Blinding was not relevant for this historical cohort study; no interventions were performed

Reporting for specific materials, systems and methods

We require information from authors about some types of materials, experimental systems and methods used in many studies. Here, indicate whether each material, system or method listed is relevant to your study. If you are not sure if a list item applies to your research, read the appropriate section before selecting a response.

Materials & experimental systems

n/a	Involved in the study
☒	☐ Antibodies
☒	☐ Eukaryotic cell lines
☒	☐ Palaeontology and archaeology
☒	☐ Animals and other organisms
☒	☐ Human research participants
☒	☐ Clinical data
☒	☐ Dual use research of concern

Methods

n/a	Involved in the study
☒	☐ ChIP-seq
☒	☐ Flow cytometry
☒	☐ MRI-based neuroimaging
